# VSOT: volume-surface optimization for accurate ultrastructure analysis of dendritic spines

**DOI:** 10.1093/bioinformatics/btaf220

**Published:** 2025-04-24

**Authors:** Boyu Lyu, Jiangxiong Wang, William Christopher Risher, Guoqiang Yu

**Affiliations:** Bradley Department of Electrical and Computer Engineering, Virginia Polytechnic Institute and State University, 900 N. Glebe Rd., Arlington, VA, 22203, United States; Beijing National Day School, No. 66 Yuquan Rd, Haidian District, Beijing, 100039, China; Department of Biomedical Sciences, Marshall University, 1 John Marshall Drive, Huntington, WV, 25755, United States; Department of Automation, Tsinghua University, No 1 Qinghuayuan Street, Haidian District, Beijing, 100084, China; IDG/McGovern Institute for Brain Research, Tsinghua University, No 1 Qinghuayuan Street, Haidian District, Beijing, 100084, China; Beijing National Research Center for Information Science and Technology (BNRist), No 1 Qinghuayuan Street, Haidian District, Beijing, 100084, China

## Abstract

**Motivation:**

Morphological analysis of dendritic spines is critical to understanding the function and dysfunction of neural circuits. The growing trends of the large-scale electron microscopy (EM) imaging systems and automatic cellular reconstruction provide unprecedented opportunities to investigate the ultrastructure of dendrites. This morphometric analysis of dendritic spines requires accurate compartment segmentation methods as well as meaningful quantification methods. However, most existing methods rely on surface or volumetric information alone, which may not deliver accurate segmentation results.

**Results:**

We developed **VSOT**, a method based on **V**olume-**S**urface **O**p**t**imization, designed for the accurate structural analysis of dendritic reconstruction. VSOT accurately segments dendritic reconstructions into compartments, including spine, spine head, and spine neck, by leveraging advanced optimization techniques that integrate local surface and global volumetric information. Our tests on public datasets of spine segmentation, as well as on a first-of-its-kind dataset of head-neck segmentation that we manually constructed, show that VSOT offers more accurate results than peer methods. When applied to a large EM dataset of different brain layers, VSOT reveals how the structure of dendrites varies across brain areas. Furthermore, we explored the structural relationships between neurons and astrocytes at tripartite synapses. With the newly developed computation methods, neuroscientists can exploit the large-scale volumetric EM data to address various scientific questions and advance the understanding of neural circuits.

**Availability and implementation:**

VSOT is available at https://github.com/yu-lab-vt/VSOT. The data and codes in this study are available at Zenodo (https://doi.org/10.5281/zenodo.15115542).

## 1 Introduction

Dendritic spines, composed of the actin cytoskeleton, are the primary sites of synaptic input in the nervous system (Harris and Kater 1994, Luo 2002). Depending on the arrangement of actin within the spine, as well as the amount of receptors, ion channels, scaffolding proteins, and other synaptic molecules present, spines take on a variety of morphologies throughout different parts of the brain. The geometry of the different compartments of the spine (i.e. the head and neck) is a critical determinant in the biophysical characteristics of these structures and thus their associated synapses (Yuste 2013). Indeed, it has been postulated that the head and neck may each play distinct roles in synaptic plasticity-related phenomena (Ofer *et al.* 2021), underscoring the importance of accurate partitioning during structural analysis. To study dendritic spines, electron microscopy (EM) is considered the gold standard in studying the ultrastructure of the brain, and particularly in deciphering the relationship between the dendritic structure and neural circuit function (Parajuli and Koike 2021, Turner *et al.* 2022, Baldwin *et al.* 2024, Seung 2024). Recent developments in EM imaging as well as EM reconstruction methods make it possible to image a large volume of the brain and obtain reconstructions of thousands of neurons and glia, such as the FlyEM dataset of drosophila central brain (Dorkenwald *et al.* 2024, Schlegel *et al.* 2024), the H01 project (Shapson-Coe *et al.* 2024), and the MICrONS (Lee *et al.* 2017, Funke *et al.* 2019, Bodor *et al.* 2021). It is imperative to design methods to accurately quantify structures like dendritic spines to deepen biological understanding. Large-scale EM datasets also present a unique opportunity to investigate the structure of the “tripartite synapse” (e.g. presynaptic axons, postsynaptic dendrites, and associated peripheral astrocyte processes) with an unprecedented level of detail. Astrocytes contact or ensheathment of synapses with widely varying frequency and coverage depending on species, developmental age, brain region, and physiological status. With the compartment segmentation of dendritic spines, we can achieve more accurate quantification of the ensheathments and tripartite contacts.

In segmenting the dendritic spines from the neuron reconstructions, researchers can manually separate the spines from the dendritic shaft (Kasthuri *et al.* 2015, Parajuli *et al.* 2020, Tamada *et al.* 2020) based on the slices of EM data. However, with the current Petabyte dataset, manual segmentation is infeasible. Automated methods for dendritic spine and shaft segmentation are typically based on morphological operations (Wildenberg *et al.* 2021, Michalska *et al.* 2024) or cylinder fitting techniques (Kashiwagi *et al.* 2019, Pchitskaya *et al.* 2023). The former approach uses erosion and dilation to remove thin spine necks, thereby isolating the dendrite shaft. However, these methods have a limited accuracy due to significant variations in spine neck thickness. The latter approaches, involving cylinder fitting, struggle to handle junction areas because of the dendrite's uneven surface. Additionally, a recent method NEURD (Celii *et al.* 2023) constructs a graph from surface over-segmentations and extracts subgraphs of shafts and spines by setting thresholds for multiple features such as length, volume, and area. However, tuning these parameters is challenging in practice.

To separate the dendritic spine head and neck, existing methods often use shape diameter or surface radius to determine the dividing line between the spine neck and head (Tamada *et al.* 2020, Ofer *et al.* 2021, Dorkenwald *et al.* 2022). However, shape diameter—a measure of the volume from each face of the surface—is limited to local spatial information. Surface radius or cross-sectional area, used by (Tamada *et al.* 2020, Dorkenwald *et al.* 2022), is not robust when dealing with tortuous dendritic spines. Additionally, generating a good surface for computation is also a problem itself, which may be the root problem of wrong calculation. A few supervised methods were designed as well. SyConn (Dorkenwald *et al.* 2017) trained a random forest classifier for classifying compartments of neuron leveraging the features such as mitochondria, vesicle clouds and synapses, which are not always available. TypeEM, used in (Motta *et al.* 2019), designed classifiers based on the superpixels in the EM data for different part of the dendrite. Although supervised methods hold great potential, their practical performance is limited, perhaps due to the lack of high-quality and large number of training samples.

**Figure 1.**
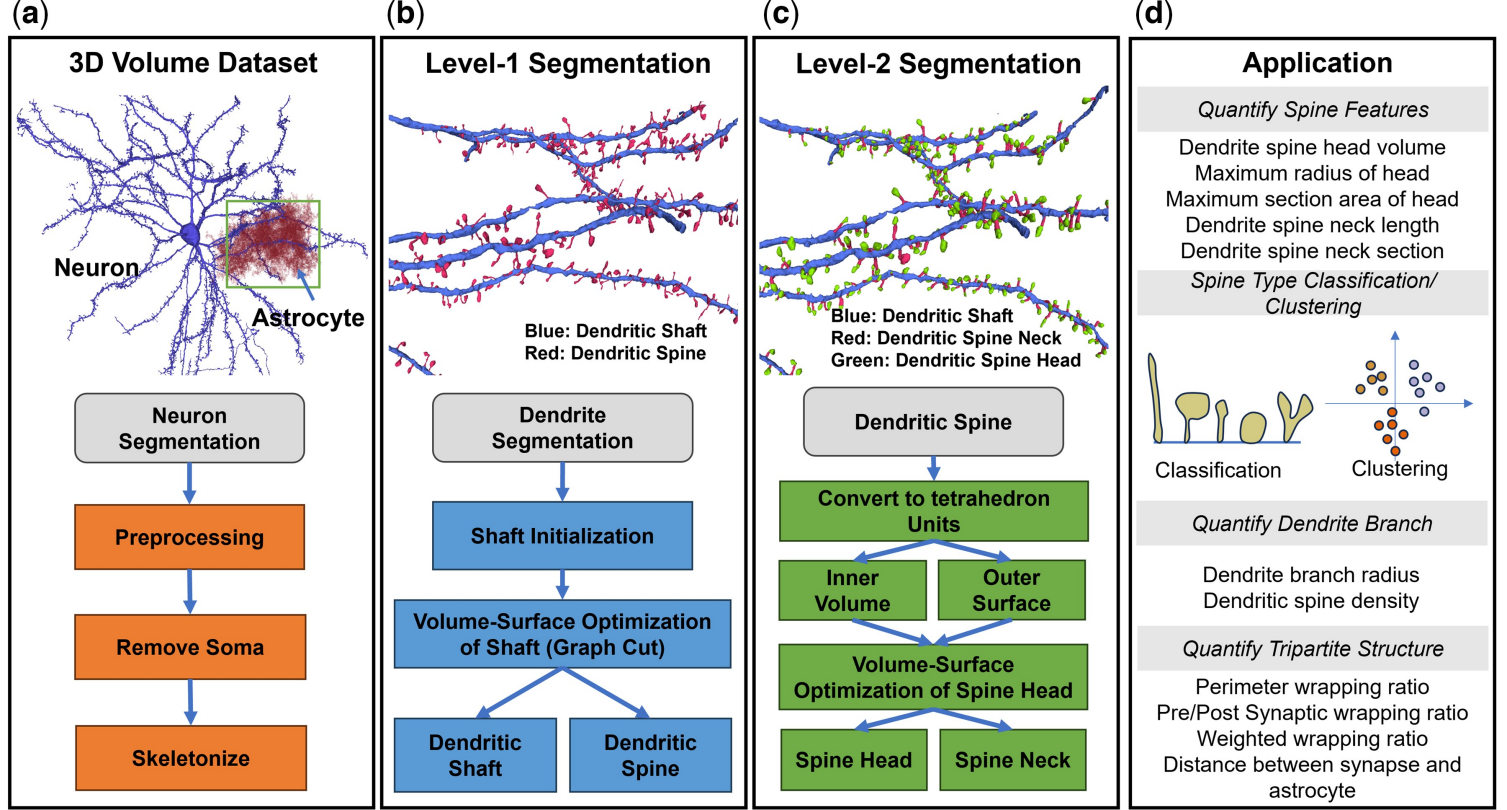
An overview of VSOT pipeline for the segmentation of dendritic spines as well as quantifications. (a) Extract the target region containing neuron/astrocyte segmentation from public EM dataset. Preprocess the segmentation to separate the dendritic branches and obtain the skeletons. (b) Level-1 segmentation of VSOT segments the dendrites into dendritic shafts and dendritic spines. (c) Level-2 segmentation of VSOT segments the dendritic spines to spine head and neck. (d) Structural quantification using the compartment segmentation of dendrites.

In this article, we designed a comprehensive pipeline for segmenting and quantifying dendritic spines. The framework is depicted in Fig. 1, with more details shown in Supplementary Fig. S1. Our approach encompasses two distinct levels of segmentation: the initial segmentation of dendritic spines (level-1 segmentation) and the subsequent segmentation of dendritic spine heads and necks (level-2 segmentation). For level-1 segmentation, we transcend traditional methods that rely solely on surface or volume data. Instead, we formulate the problem as a global optimization that seamlessly integrates both volumetric and surface modeling. This novel strategy not only ensures a smooth separation between the dendritic spine and shaft but also adeptly handles surface irregularities, such as bumps on the dendritic shaft. In the level-2 segmentation, which separates the dendritic spine head from the neck, we again built the model using both surface and volumetric features. In our method, rather than simply relying on the dividing boundary, we further reasoned that the extracted head region is expected to approximate a sphere, which is commonly observed but rarely incorporated into modeling in previous studies. Incorporating this shape information greatly elevates the robustness of the segmentation.

To test the performance of VSOT, we curated new datasets with manually generated ground truths. Our comparative analysis demonstrates that our method outperforms existing state-of-the-art techniques, offering more accurate and robust segmentations which arise from the volume-surface optimization formulated for both tasks. This framework of two-level segmentation can help biologists delve into large-scale EM datasets to analyze tripartite structures with an unprecedented degree of accuracy. As an application, we showcased the structural analysis of dendritic spine and tripartite structures by applying our pipeline to an EM dataset containing multiple cortex layers.

Our proposed framework was based on four major technical innovations. (i) Integration of volume and surface features: We incorporated both volumetric and surface characteristics into modeling of the segmentation of dendritic spines, as well as segmenting the spine head and neck. This dual-feature approach enhances segmentation accuracy and robustness. (ii) Global optimization solved via graph theory: by formulating the segmentation problems as global optimization tasks and solving them using graph theory, we achieved efficient and optimal solutions. (iii) Bridging computational spaces with tetrahedron mesh: a new computational unit was designed to bridge surface and volumetric spaces, enabling seamless integration and improved robustness in our segmentation pipeline. (iv) Novel quantitative metrics for structural analysis: we introduced new scores for the structural quantification of dendritic spines and tripartite structures, providing deeper insights into dendritic morphology.

## 2 Materials and methods

Each individual dendritic branch is assumed to be given or can be obtained by removing soma from the dendrite structure as discussed in Supplementary Fig. S2a. For each branch, we then apply level-1 and level-2 segmentations sequentially to obtain the compartment segmentation.

### 2.1 Level 1: separating dendrite shaft and spine

For the segmentation of dendrites into dendritic shafts and spines, due to the significant variability among dendritic spines, traditional methods struggle to effectively separate the dendritic shaft from the spine when relying solely on volumetric information like distance measures or erosion techniques. Surface segmentation methods that identify cycles surrounding the spine root are similarly limited as the local region of surface is sensitive and error prone. To overcome these challenges, we introduce an innovative approach that integrates both surface and volumetric information. We expect that, for a good segmentation of spines, the dendritic shaft should maximize its volume along its backbone and minimize at the same time its surface area. In this way, on one hand the spines can be removed from the dendrite so that the resulting dendritic shaft has a minimum surface. On the other hand, the irregularities on the surface of dendritic shaft, such as small bumps, can be tolerated, because including the small bumps into the shaft tends to increase the volume but not decrease the surface area much.

Combining the two objectives, we can formulate the objective function as,

(1) minVs⁡Surface(Vs)-λ×Volume(Vs)

where we denote the voxel set in the whole dendrite as V, the voxel set of the dendritic shaft defined as $V_{s}\in V$.The term $\mathrm{Surface}(V_{s})$ is defined as the number of voxels on the outer surface of the dendritic shaft, while $\mathrm{Volume}(V_{s})$ is defined as number of voxels in the dendritic shaft. The parameter λ controls the balance between surface and volume, such that, the smaller it is the smoother the surface of the dendrite shaft, but at the same time, the volume of the dendrite shaft will shrink, leading to inseparability between spines. Otherwise, with a very large λ, dendritic spines cannot be segmented completely. This balance allows us to achieve a smooth cut while accommodating minor surface irregularities, leading to more accurate and robust segmentation results.

To solve this optimization problem efficiently, we convert it into a *s*–*t* graph cut problem as shown in Supplementary Fig. S2b. In our graph design ={V, E}, voxels are defined as nodes, with each pair of neighboring voxels i, j connected by an edge $e_{ij}.\;$Additionally, we connect all the voxels on the dendrite surface to the pseudo sink node $e_{jt}.$ The cut resultant from the edges $e_{ij}\;$will contribute to the calculation of the $\text{Surface}(V_{s})$ term in Equation (1). Finally, we connect all the voxels to the pseudo source node $e_{si}$, cutting which will contribute to the outer volume of $V_{s}$, $\lambda \times (\mathrm{Volume}(V)-\mathrm{Volume}(V_{s}))$. Minimizing this component corresponds to minimizing the second term in Equation (1). In this way, Equation (1) can be solved by finding the min source-sink cut. More details can be found in Supplementary S2.1.

### 2.2 Level 2: segmenting dendritic spine head and neck

Before segmenting the dendritic spine head and neck, we first filtered out the branched spines because of the challenge of separating them into individual head and neck compartments for the structural analysis. Thus, we focused on the segmentation for non-branched dendritic spines. Since the shape of dendritic spine is normally described as a bulbous spine head on top of a thin neck (Nimchinsky *et al.* 2002), defining the optimal dividing plane between the dendritic spine head and neck is a central challenge in level-2 segmentation. Existing methods often rely on surface curvature, skeletal structures, or cross-sectional diameters to detect the dividing line between head and neck, without incorporating the spherical shape of the dendritic spine head. By neglecting this shape information, these methods may be highly sensitive to the uneven surface present on spines. We therefore combined the shape information (global volumetric information) with local surface information for a more robust segmentation.

In our model, we assume that the optimal cut goes through the vertex $i\in V_{\mathrm{surface}}\;$on the surface. We modeled that the point i should locate close to a saddle point on the surface, and at the same time, the dendritic spine head should also be spherical. Gaussian curvature is an intrinsic measure of the saddle point, defined as $K=k_{1}k_{2}$, with K<0 representing a saddle point. The smaller this score, the more likely the point is desired. On the other hand, to measure the sphericity of the head, we defined a geometry score $S_{i}$ for the voxels in the head. The volume of the head if cut at point i is defined as ${\mathrm{Vol}}_{i}\;$and the surface of the head is defined as ${\mathrm{Area}}_{i},$ then we define the sphericity $S_{i}\;$as,

(2) Si=rA(i)-rV(i)rA(i) 

 

(3)  rA(i)=Areai4π and rV(i)=3×Voli4π3

where $r_{A}(i)$ is the radius of the equivalent sphere with the same surface area, and $r_{V}(i)$ is the radius of an equivalent sphere with the same volume.

**Figure 2.**
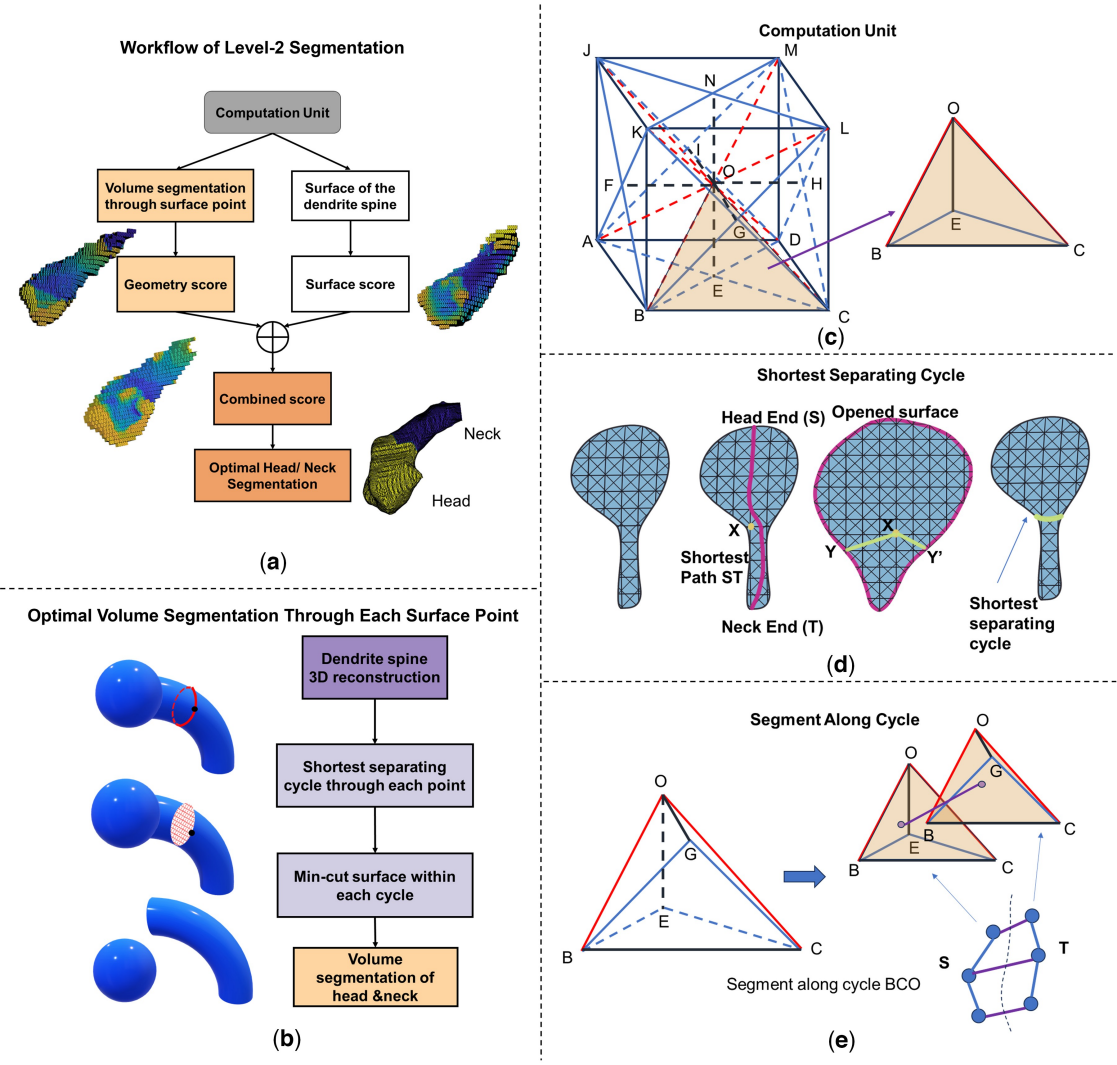
(a) Workflow of the level-2 segmentation. (b) Workflow of obtaining the volume score for each surface point. (c) We define the computation unit using tetrahedrons with voxels as vertices. This way, we bridged the computation between the surface and the volumetric space. (d) In obtaining the shortest separating cycle, we opened the surface of dendritic spine and then search for the shortest path between the target point Y and its dual Y’. (e) We built a graph of computation units as nodes, and the overlapping triangle area as edge weight between nodes. By finding the min-cut in the graph, we can find the min-cut surface between head and neck.

Among all the objects with different shapes, the sphere has the smallest surface-to-volume ratio (S2V), which means the structures that are close to the sphere will have a smaller $S_{i}.\;$Thus the overall objective is,

(4) mini⁡f(Si)+g(Ki)+λR(Si,Ki) 

where $f(S_{i})$ and $g(K_{i})$ are functions to ensure the compatibility when combining two scores, while $R(S_{i},K_{i})$ is the regularization function of the two scores. The difficulty in optimizing Equation (4) is the chicken-and-egg problem. To get a good segmentation, we need to assess the sphericity using geometry score $S_{i}$. At the same time, to assess the sphericity, we need to get the candidate segmentation first. However, there are many possible candidate segmentations through a certain surface point, which makes the problem intractable. We observed that for each surface point a minimum cutting plane with a smooth surface can be obtained so that the resultant segmentation has a good sphericity score. In fact, it can be shown that the resultant segmentation is the most spherical passing through the point for a cylinder. In other words, we can define an upper-bound sphericity for each surface point. Therefore, instead of optimizing the sphericity and surface curvature directly, we now aim to optimize the upper-bound of the sphericity and the surface curvature as shown in in Fig. 2a.

For each point on the surface, to obtain its optimal head segmentation with good sphericity score, we define the cut plane to be minimum area. Since we need to obtain the candidate for each point, efficiency is one critical factor in designing the method. Instead of directly searching the minimum-area cut for each point using the whole volume of dendritic spine, we first obtain the shortest separating cycle through the given point on the surface as initialization, then we find the head-neck cut with minimum area inside the cycle, as shown in Fig. 2b. By searching for the min-surface cut within a small volume with the constraint of the border polygon, we reduce the computation load. Using graph theory, we formulated global optimization for both tasks. After obtaining the candidate head region for each surface vertex, a unified score for each candidate was designed by involving both the surface and volumetric features. With this combined score map, we search for the optimal cut for final segmentation.

Before introducing our volume-surface optimization model for the two aforementioned tasks, since the final output are voxel sets for the dendritic spine head and neck, it is crucial to maintain consistency between the surface and volume representations during computation. However, typical mesh-generation methods create surfaces that may significantly deviate from the volume's outer voxels. To bridge the two spaces to ensure consistency, we designed a new computation unit that enforced the vertices on the regular grid points.

#### 2.2.1 A new unit for both volume and surface computation

Our newly designed computational unit is based on the tetrahedron volumetric mesh. As shown in Fig. 2c, for a cubic region with one center voxel together with its 14 neighbor voxels, we can split them into 24 tetrahedrons, 4 per face. Each tetrahedron will be denoted as a computation unit containing both the neighboring information and the surface area information. Since these computation units have real voxels as terminals, this aligns the computation in both surface and volumetric space.

#### 2.2.2 An efficient method to obtain the shortest separating cycle through a surface point

The intersection between the dendritic spine neck and the dendritic shaft, obtained through level-1 segmentation, is denoted as the neck end points of the dendritic spine. The point in the dendritic spine that is furthest from neck end is denoted as the head point. The goal is to find the shortest cycle that cuts the surface between the head end and the neck end. A straightforward way is to obtain a s-t min-cut constraining the cut through the target point. This can be achieved by constructing a graph with each vertex on the surface as nodes and edges of triangles as graph edges, with two ends of the dendritic spine as source and sink nodes separately. However, the graph has to be reconstructed for each target surface point to constrain the cut through the target point. Since min-cut requires $O(N^{3})\;$time-complexity for each surface point, where N represents the total number of surface points, the total time-complexity for this naïve design would be $O(N^{4})$. Inspired by Reif's algorithm (Reif 1981), we convert this min-cut problem of an open-disk graph into a more efficient shortest path algorithm in the dual planar graph. This is done by splicing the 3D graph through a path linking the two end points of the dendritic spine and the target points (Fig. 2d). After splicing, the path will be duplicated, as will the target point x, which we denote its twin as x′. We can then obtain the shortest separating cycle by searching for the shortest path between x and x′, which allows us to reduce the total complexity from $O(N^{4})$ to $O(N^{2})$. Details on complexity reduction are in Supplementary Material S2.2.

#### 2.2.3 Min-area cut given a bounding cycle

The next objective was to determine the minimum surface cut enclosed by the shortest separating cycle. Finding the minimum surface given a boundary lies in the category of the renowned Plateau problem, but the majority of the study was based on the smooth boundary or the piece-wise boundary (Pinkall and Polthier 1993, Arnal *et al.* 2003). Other than the condition of smooth boundary, various methods have been designed to obtain the triangle meshes with the minimum area given a polygon boundary. For instance, Chen *et al.* (2008) introduced one iterative method to update an initial mesh by minimizing the Dirichlet integral. Liu (2011) introduced another iterative method with slightly lower error for a sparse set of points based on minimizing the mean curvature. However, both methods require the input of an initial mesh; in addition, they cannot guarantee a global minimum.

To address these challenges, especially in the context of real-world applications involving volume segmentation, we initialize the dendritic spine volume using our newly designed computational units, all tetrahedrons. This approach enables us to formulate the aim as to identify the set of triangles in these tetrahedrons that are encircled by the separating cycle and, together, have the minimum total area. It follows that as the grid becomes infinitely fine, each triangle in the optimal triangulation can be approximated by smaller triangles formed by the grid points, making the approximation error negligible. Thus, finding the optimal set of triangles is asymptotically the best triangulation with the global minimum surface.

In our graph design, each node corresponds to one computation unit (tetrahedron), as shown in Fig. 2e; if there were a triangle shared by any pair of adjacent tetrahedrons, two corresponding nodes are linked by an edge. The edge weight is defined as the area of the shared triangle. Thus, by defining the source node and sink node as the two ends of the dendritic spine, the minimum *s*–*t* cut directly gives us the solution to the target problem. More algorithmic details are in Supplementary Material S2.3.

#### 2.2.4 Search for the optimal surface point

Using the segmentation obtained from each point on the surface, we designed scores quantifying both its surface feature and geometry feature: (i) surface score $S_{i}$: the Gaussian curvature at each point on the surface; and (ii) Geometry score $K_{i}$: the sphericity of the head region after separating the spine at a certain point with the minimum cut plane.

For the surface feature, since the curvature is sensitive to local change, we first applied Taubin smoothing (Taubin 1995) to smooth the surface without shrinkage. In addition, to adapt to different resolutions of data, the scale needs to be adapted to generate the optimal score map for identifying the bend-shape at the optimal dividing cycle. We designed a way to calculate the curvature with tunable window size (nm), which could be modified based on the resolution of the EM data, as described in Supplementary Material S2.4. An example of the surface score is in Supplementary Fig. S3.

Since both scores $S_{i}$ and $K_{i}$ are calculated for each vertex on the same triangulation surface, they can be matched vertex-wise. The smaller the score value, the better the segmentation. To ensure the two scores can be combined at the same scale, we converted both scoremaps to the map of ranks. Then we defined the combined scoremap as the sum of the rankmap of two scores:

(5) Cirank=Sirank+Kirank

The optimal point for separation was defined with the smallest combined score. Then, we could separate the dendritic spine into two parts by first finding the shortest separating cycle and then the min-surface cut, as mentioned in Sections 2.2.2 and 2.2.3.

## 3 Results

### 3.1 Datasets

We curated a dataset for evaluating the level-1 segmentation from (Kasthuri *et al.* 2015). Due to the sparse labelling of dendrites, we selected the largest one with one apical dendrite and five basal dendritic branches, which is the dendrite number 5 from the annotation, as shown in Supplementary Fig. S4. We converted the .obj files into volumetric data generate test samples for level-1 segmentation, yielding 344 dendritic spines.

For level-2 segmentation and the structural analysis of dendritic spines and tripartite interactions in multiple cortical layers (Sections 3.4–3.6), we first selected 23 astrocytes from layers 2/3, 4, and 5 from the MICrONS dataset, as shown in Supplementary Fig. S6a. These astrocytes were chosen based on manual verification of 3D reconstruction completeness. We then automatically selected the top 30 neurons with the largest soma-free volume surrounding each astrocyte and retained those with branches predominantly localized within astrocyte regions. Additionally, we focused on excitatory pyramidal neurons and excluded inhibitory neurons. This process resulted in 362 neurons (L2/3: 123, L4: 129, L5: 110) across the three layers with details in Supplementary Data. Then we applied level-1 segmentation on this dataset and obtained in total 61 117 dendritic spines (L2/3: 22807, L4: 23610, L5: 14700).

From each layer, we uniformly selected 300 dendritic spines and removed erroneous segmentations. Since filopodia-shaped dendritic spines are usually ignored in the counting due to their limited numbers (Pchitskaya and Bezprozvanny 2020), accounting for only 3.4% of spines in this dataset, we also removed these in the ground truth dataset. To ensure enough samples for different spine types (thin, mushroom, and stubby), we included additional 67 stubby spines for performance testing. Various types of dendritic spine are shown in Supplementary Fig. S6b. Three trained annotators independently labeled the samples under expert supervision. To obtain a reliable segmentation from the three annotators, we checked the consistency between the cut locations from each pair of annotators. We used the Intersection over Union (IOU) to measure how well the two cuts are aligned and applied two thresholds ($T_{1}$ and $T_{2}$) for filtering, such that, if the IOU of three pairs are all larger than $T_{1}=0.9$, then we considered the three aligned. When two pairs are not aligned, but the third IOU exceeds $T_{2}=0.95$, the corresponding pair will also be deemed aligned. In total, we generated consistent annotations for 703 out of 767 samples.

### 3.2 Performance evaluation on level-1 segmentation

**Figure 3.**
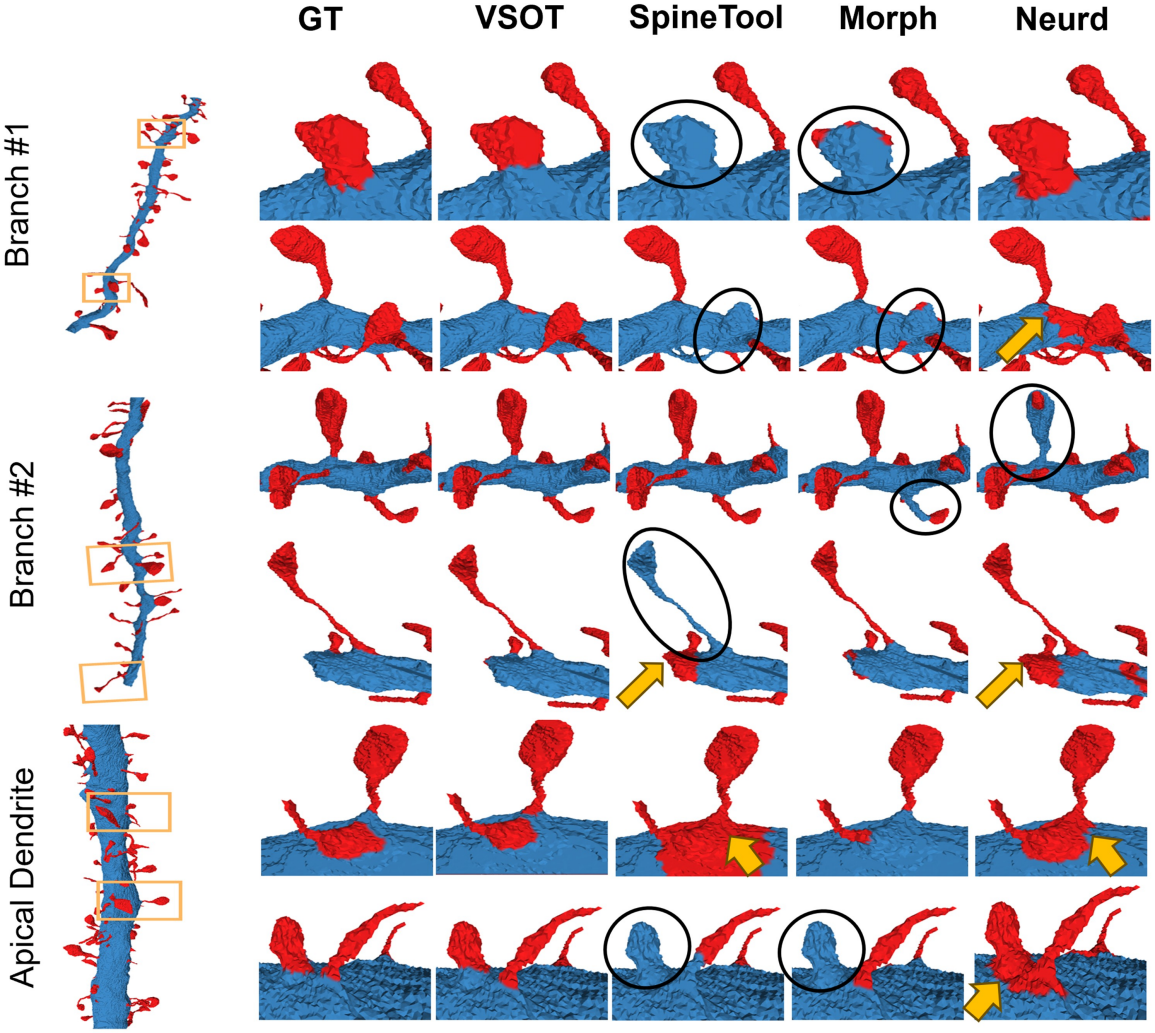
Visualization of the performance for level-1 segmentation of dendritic spines (red) emerging from shafts (Blue). Black circles indicate the false negatives. The arrow indicates the error that the segmentation of the spine is larger than the ground truth.

Three state-of-art methods were chosen for the comparison, including two surface segmentation methods, NEURD (Celii *et al.* 2023) and SpineTool (Pchitskaya *et al.* 2023), and a volumetric segmentation method (Michalska *et al.* 2024) (referred to as “Morph” hereafter). We used precision, recall, F1-score and IOU to measure performance. Precision, recall and F1-score are defined as Precision=TP/(TP+FP), Recall=TP/(TP+FN) and F1=2×(Precision×Recall)/(Precision+Recall), where TP, FP, FN are calculated by comparing to the ground truth. As shown in Table 1, VSOT outperformed all peer methods for all four criteria. We found that the surface segmentation method, SpineTool (Fig. 3, third column), tends to miss small dendritic spines. The volume segmentation method (Morph) using morphological operations works well in certain cases where the dendritic shafts are remarkably thicker than the dendritic spine. However, when their thickness is close, this method can miss many detections (Fig. 3, fourth column). While NEURD does not show a consistent trend (Fig. 3, fifth column): for certain samples, it is prone to generate more false positives, while for others it is biased the opposite direction. More details are in the Supplementary Material S3.1 and Supplementary Fig. S5.

**Table 1.** Performance testing results for level-1 segmentation (bold values indicate the highest scores among the four methods).

	F1-score	Precision	Recall	IOU
VSOT	**0.953 ± 0.016**	**0.963 ± 0.017**	**0.941 ± 0.019**	**0.746 ± 0.173**
SpineTool	0.888 ± 0.079	0.891 ± 0.109	0.889 ± 0.053	0.630 ± 0.110
Morph	0.882 ± 0.071	0.954 ± 0.105	0.822 ± 0.046	0.668 ± 0.224
Neurd	0.808 ± 0.034	0.718 ± 0.024	0.935 ± 0.054	0.386 ± 0.108

### 3.3 Performance evaluation on level-2 segmentation

**Figure 4.**
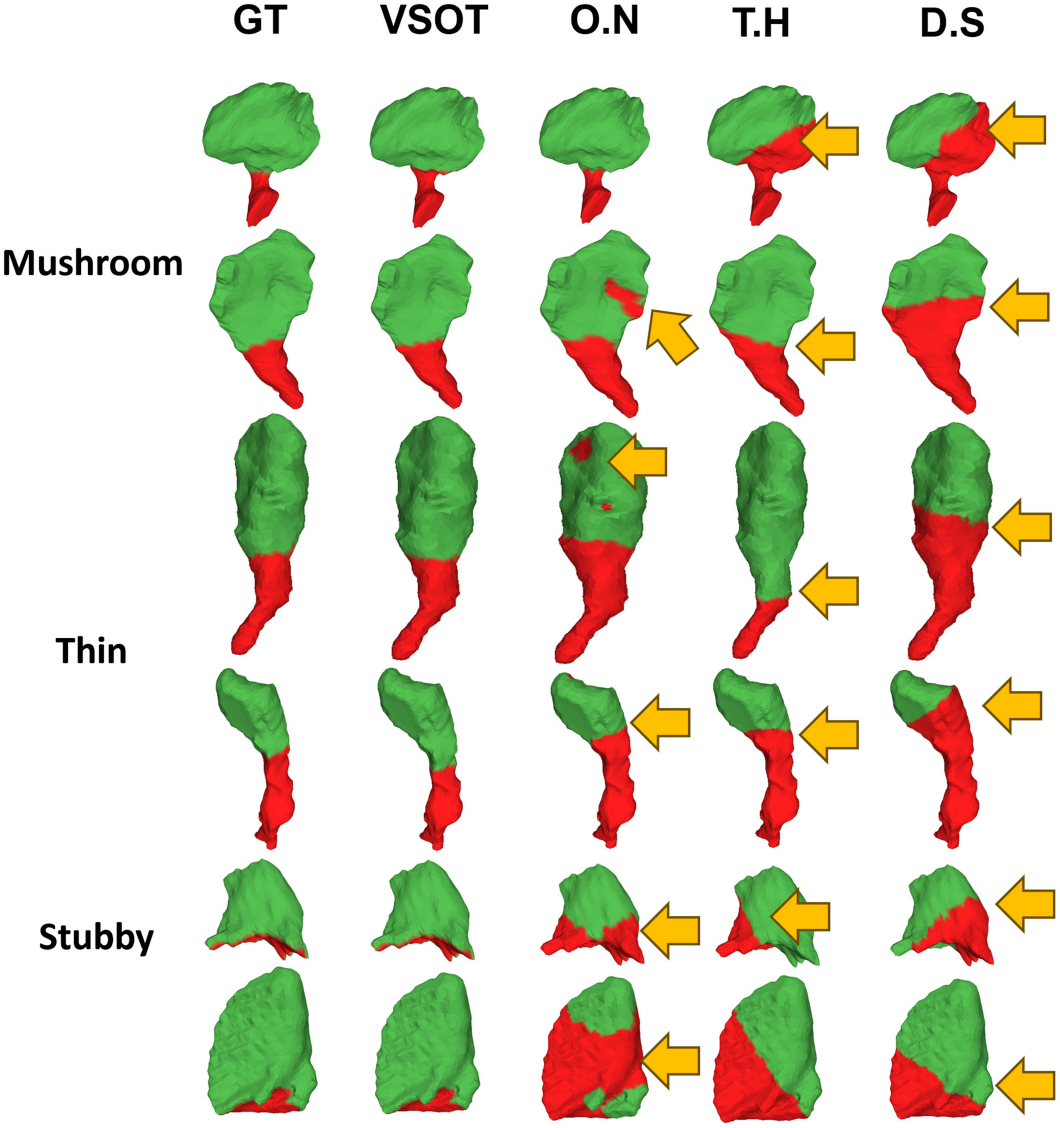
Dendritic spine head (green)/neck (red) segmentation results from VSOT compared with three peer methods.

VSOT was compared with three methods that were designed for segmenting the spine head and neck: O.N method (Ofer *et al.* 2021), D.S method (Dorkenwald *et al.* 2022), and T.H method (Tamada *et al.* 2020). VSOT’s results are the closest to ground truth (Fig. 4), maintaining the features such that the spine head is close to a spherical structure, with the division happening when there is a sharp change on the surface. In addition, VSOT provides more robust and consistent results when dealing with spines with different shapes. Regarding the three peer methods, surface segmentation cannot give robust results due to surface noise. In addition, the heuristics designed in the D.S and T.H methods cannot handle the variability of the shape of the spine. Furthermore, the cross-section area used in T.H. cannot capture the shape change, which leads to increased errors. To quantitatively measure the performance, we used precision, recall, F1 score defined similar to the metric used in level-1 segmentation, as well as IOU. Specifically for the first three scores, we define the TP, FP, FN based on the segmentation of dendritic spine head. As in Table 2, VSOT outperformed peer methods in terms of F1-score, Recall and IOU. D.S method can be better in terms of precision, which is largely due to its separation cycle which tends to move closer to the head part. Since precision measures only spine segmentations, D.S tends to have better precision. However, the recall of D.S is poor, resulting in an overall worse performance than VSOT.

**Table 2.** Quantitative results of our level-2 segmentation method compared to peer methods (bold values indicatethe highest scores among the four methods).

Metrics	VSOT	O.N	T.H	D.S
Mushroom dataset				
F1	**0.93 ± 0.10**	0.88 ± 0.20	0.84 ± 0.17	0.77 ± 0.15
Precision	0.94 ± 0.13	0.93 ± 0.19	0.93 ± 0.16	**0.97 ± 0.13**
Recall	**0.94 ± 0.13**	0.85 ± 0.21	0.81 ± 0.21	0.66 ± 0.19
IOU	**0.91 ± 0.11**	0.86 ± 0.17	0.81 ± 0.16	0.74 ± 0.14
Thin spine dataset				
F1	**0.90 ± 0.11**	0.80 ± 0.26	0.82 ± 0.18	0.76 ± 0.13
Precision	0.92 ± 0.16	0.89 ± 0.24	0.91 ± 0.17	**0.97 ± 0.12**
Recall	**0.91 ± 0.11**	0.78 ± 0.27	0.81 ± 0.22	0.65 ± 0.18
IOU	**0.87 ± 0.12**	0.87 ± 0.20	0.80 ± 0.15	0.74 ± 0.14
Stubby spine dataset				
F1	**0.84 ± 0.22**	0.59 ± 0.35	0.59 ± 0.29	0.72 ± 0.20
Precision	**0.94 ± 0.22**	0.84 ± 0.26	0.87 ± 0.25	0.91 ± 0.23
Recall	**0.79 ± 0.23**	0.52 ± 0.35	0.50 ± 0.30	0.63 ± 0.20
IOU	**0.80 ± 0.20**	0.56 ± 0.30	0.55 ± 0.24	0.64 ± 0.17
Combined dataset				
F1	**0.92 ± 0.13**	0.83 ± 0.25	0.81 ± 0.21	0.76 ± 0.16
Precision	0.93 ± 0.15	0.91 ± 0.21	0.92 ± 0.17	**0.97 ± 0.14**
Recall	**0.92 ± 0.13**	0.80 ± 0.26	0.78 ± 0.25	0.65 ± 0.19
IOU	**0.89 ± 0.13**	0.82 ± 0.21	0.78 ± 0.19	0.73 ± 0.15

### 3.4 Quantifying dendritic spines across cortex layers

**Figure 5.**
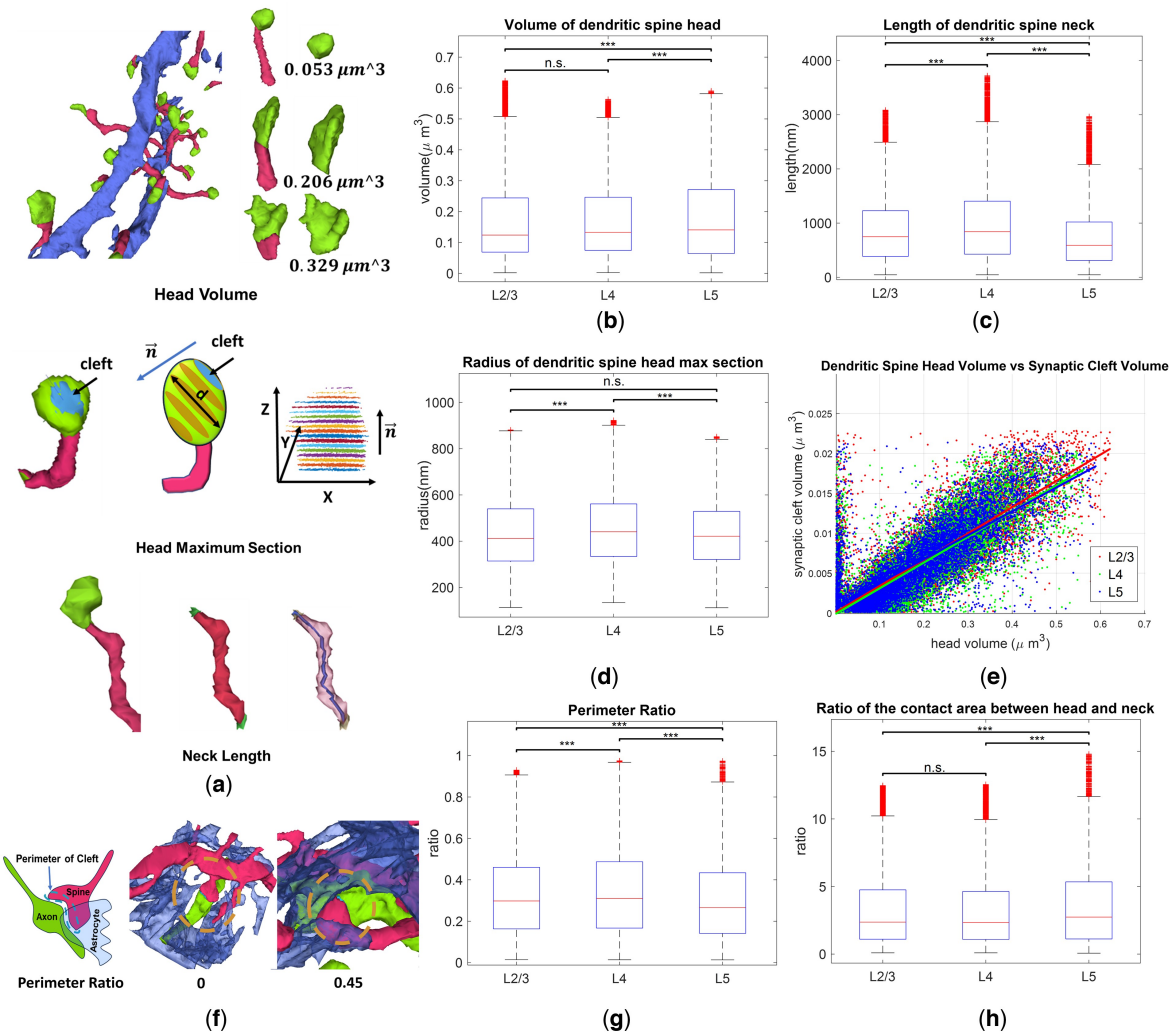
(a) Illustration and example of quantifying dendritic spine structures. With the head, neck segmentation, we can quantify the features like head volume, max section of head, neck length, etc. (b) Head volume in layer 5 is larger than the other two layers. (c) The length of spine neck in layer 4 is significantly longer than the other two layers. (d) The radius of the maximum head section in layer 4 is significantly larger than the other two layers. (e) Linear relationship between the dendritic spine head volume and PSD size. (f) An example of the parameter ratio quantifying how well the astrocyte wraps around the cleft’s perimeter for the tripartite structure. (g) The perimeter ratio is significantly larger in layer 4. (h) The ratio between the head/neck contact area is significantly larger in layer 5.

Using our two-level segmentation approach on these samples, we identified over 30 000 dendritic spines. Upon segmenting these compartments (Fig. 5a), we developed scoring metrics to quantify their structures, such as spine volume, spine head radius, and spine neck length (Supplementary Material S4).

Our large-scale analysis revealed significant heterogeneity among the layers. The length of the spine neck was significantly higher in layer 4 compared to the other two layers (Fig. 5c, L2/3, L4, L5, N: 20063, 20927, 12343. Mean (nm) ± SE: 874.9 ± 4.30, 1001.3 ± 4.99, 734 ± 4.94, *t*-test). The analysis of spine neck radius (Supplementary Fig. S9b) indicates that layer 4 spines also have a larger proportion of thicker necks. These results are in line with previous research (Sun *et al.* 2016) suggesting that the unique spine morphology of layer 4 could be due to its dual role in receiving signals from both adjacent layers and the thalamus, necessitating adaptations for a larger receptive field. In addition, we confirmed the correlation between spine head volume and the volume of post-synaptic density, as shown previously (Konur *et al.* 2003) (Fig. 5e).

Spine head volume, illustrated in Fig. 5b (L2/3, L4, L5: N: 22071, 22862, 14195, Mean ($\mu m^{3}$)±SE: 1.67e−01 ± 8.85e−04, 1.68e−01 ± 8.08e−04, 1.77e−01 ± 1.1e−03, *t*-test) shows that the mean of the spine head volume was significantly larger in layer 5 than either layer 2/3 or layer 4, consistent with previous findings (Konur *et al.* 2003). Supplementary Figure S9a revealed a bimodal pattern in layer 5, suggesting a higher prevalence of mushroom-like spines. Interestingly, when examining the maximum head section parallel to the clefts, layer 4 shows a significant larger radius than the other two layers with a smaller volume (Fig. 5d, N: 15217, 14676, 9047, Mean (nm)±SE: 4.34e+02 ± 1.23, 4.56e+02 ± 1.28, 4.32e+02 ± 1.48, *t*-test), which might indicate the structure of head in layer 4 to be more polarized. We also investigated if different layers contained distinct types of dendritic spines: specifically, whether they were spiny or filopodia-like. As in Supplementary Fig. S9c, no significant difference was found between layers.

### 3.5 Structural analysis of tripartite synapses

One key function of the astrocyte is to uptake glutamate from the extracellular space through transporters including excitatory amino acid transporter 1 (EAAT1) and EAAT2. It is hypothesized that these transporters are predominantly located just outside the synaptic cleft to maximize the astrocyte’s sensitivity to changes in glutamate spillover, given this region's high dynamic response range. As a measurement of tripartite structure, we assessed the extent of astrocyte envelopment of the synapse (Fig. 5f), such as the perimeter contact ratio measuring how well the astrocyte wraps around the synaptic cleft region (more score designs can be found in the Supplementary Materials S6, Supplementary Fig. S10).

The perimeter contact ratio in layer 4 is significantly larger than that in the other two layers (Fig. 5g, N: 12469, 11512, 6086, Mean±SE, 3.29e−01 ± 1.9e−03, 3.46e−01 ± 2.1e−03, 3.08e−01 ± 2.7e−03, *t*-test). Similar patterns can also be observed in evaluating the astrocytic contact ratio at the pre- and post-synapse regions (Supplementary Fig. S11a and b). Since the pyramidal neurons in layer 5 can connect to more distant brain regions, such as the thalamus and motor cortex, we expect layer 5 to have an intricate signal diffusion, which requires more astrocytic interplay than shallower layers. However, the astrocyte contact ratio in layer 5 is significantly lower than the other two layers, which might be attributed to the lower density of astrocytes (GFAP-immunopositive) (Forrest *et al.* 2023) or pyramidal neurons (Tjia *et al.* 2017) in layer 5. Additionally, comparing layers 2/3 and 4, the complex and high traffic in the neuron circuit of layer 4 requiring a much more frequent interplay with astrocytes might be the reason of a higher coverage by astrocyte processes.

We also examined the ratio between astrocyte contact area at the spine head and neck (Fig. 5h). Among the three layers, layer 5 showed the largest difference (L2/3, L4, L5, N: 8900, 8197, 3881, Mean±SE: 3.23 ± 0.031, 3.26 ± 0.032, 3.76 ± 0.055, *t*-test), which might be attributable to the increased head volume in this layer. This might also come from the shorter neck length in layer 5 (Fig. 5c), which leaves limited space for astrocyte processes to contact.

### 3.6 Classification and clustering of dendritic spines based on the spine head and neck quantification

**Figure 6.**
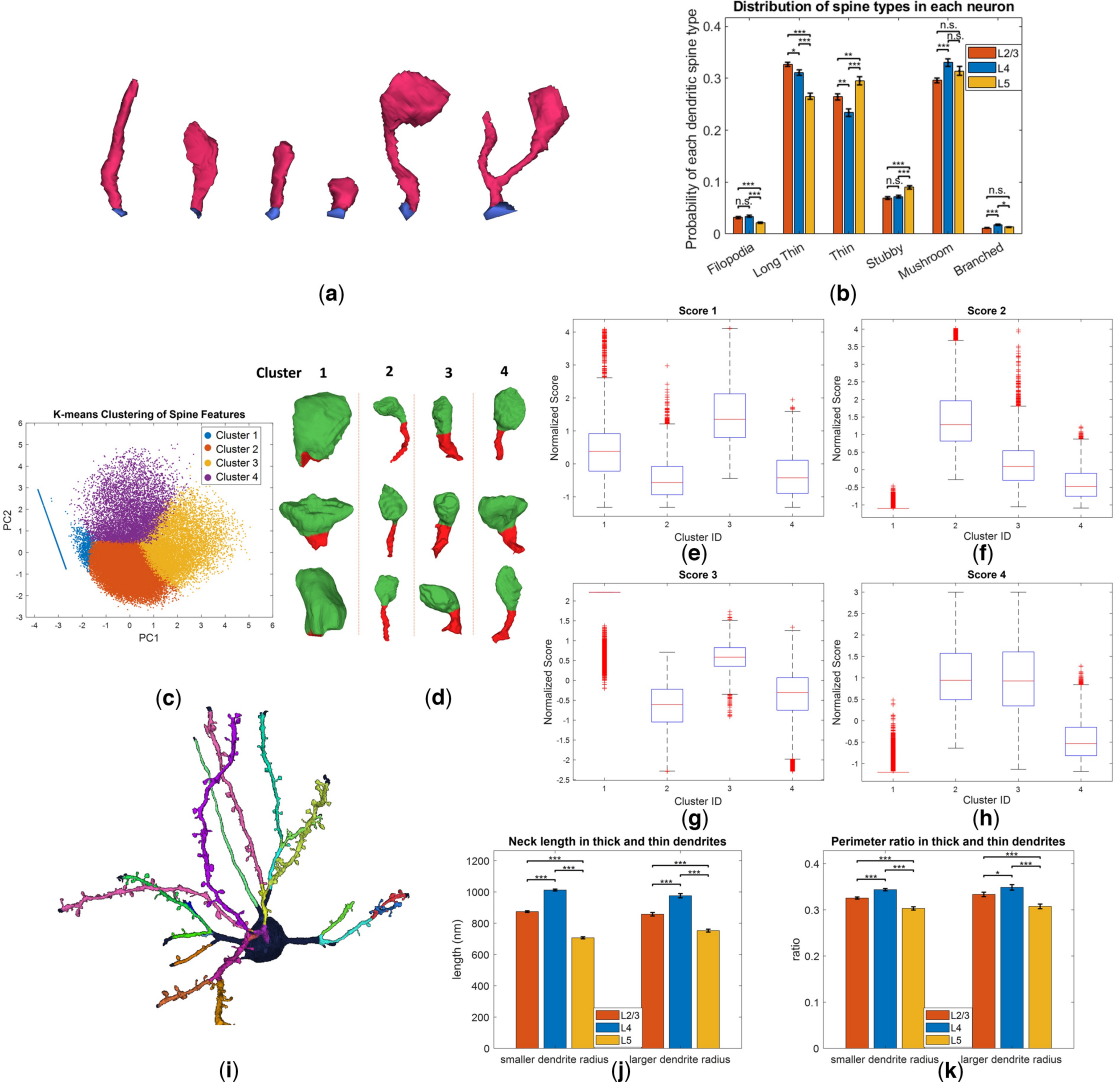
(a) Classification of dendritic spines into six types, filopodia, long thin, thin, stubby, mushroom, and branched. (b) Distribution of dendritic spines per neuron across different layers. (c) Clustering of dendritic spines using customized designed features. Different clusters encompass different shapes of spines. The cluster 1 is uniquely separated from the others because it contains mostly stubby samples without neck. (d) The difference between clusters can also be reflected from the mean scores in each cluster(e–h). (i) Split of the dendrite into individual branches. (j) After categorizing dendrites as being either thick or thin, results of mean spine neck length and (k) astrocyte perimeter ratio.

Much research on dendrites focuses on the classification of dendritic spines (Luengo-Sanchez *et al.* 2018, Pchitskaya and Bezprozvanny 2020, Pchitskaya *et al.* 2023). With our quantification of the dendritic spine head and neck, we also classified the dendritic spines into groups, following criteria similar to those outlined by (Risher *et al*. 2014) (Supplementary Materials S9, Supplementary Fig. S13). These categories include branched, filopodium, long thin, thin, stubby, and mushroom spines (Fig. 6a), representing various stages of spine maturity and synaptic strength. The distribution of dendritic spine types at different layers shows that more superficial layers tend to have a higher proportion of long thin or thin dendritic spines [Fig. 6b, N (L2/3, L4, L5):123, 129, 110 Mean ± SE, from left to right L2/3, L4 and L5, Filopodia: 0.032 ± 0.0016, 0.034 ± 0.0018, 0.022 ± 0.0014, Long Thin: 0.327 ± 0.0043, 0.311 ± 0.0051, 0.266 ± 0.0054, Thin: 0.264 ± 0.0058, 0.234 ± 0.0073, 0.295 ± 0.0080, Stubby: 0.069 ± 0.0028, 0.072 ± 0.0029, 0.090 ± 0.0037, Mushroom: 0.296 ± 0.0047, 0.331 ± 0.0071, 0.314 ± 0.0086, Branched: 0.012 ± 0.0009, 0.018 ± 0.0014, 0.013 ± 0.0011. Mann–Whitney *U* test]. Moving deeper into the brain, the likelihood of the larger mushroom spines increases.

Other than the fixed categories that we classified, in accordance with the continuum-like nature of spine structures, we further developed five scoring metrics to apply clustering on the spine structures from each layer. Which are (i) $1/r_{\mathrm{head}}$ and (ii) $l_{\mathrm{neck}}/r_{\mathrm{neck}}^{2}$, both are inspired by the formula for electric resistance in terms of length and radius. (iii) $\exp(-r_{\mathrm{head}}/r_{\mathrm{neck}}),\;$an exponential decay function inversely related to mushroom-shaped spines (smaller values indicate higher likelihood that the dendritic spine is mushroom shaped.) (iv) $l_{\mathrm{neck}}/r_{\mathrm{head}}$, the larger this score, the more likely it is close to a filopodia shape. (v) Neck cylindricity, defined as the standard deviation of $r_{\mathrm{neck}}$, quantifying variability in neck radius. As shown in Fig. 6c–h, the dendritic spines can be clustered into several groups, but the continuity between clusters makes it hard to separate them clearly.

### 3.7 Structural quantification of dendrites at different visual cortex layers

Building on the level-1 segmentation and quantification results from the previous sections, we further examined the heterogeneity of dendritic structures across different visual cortex layers by assigning individual labels to each dendritic branch (Fig. 6i, Supplementary Materials S8 and Supplementary Fig. S12). Unlike the previous subsections that focused on the structure of dendritic spines, this section studies the broader dendritic structure.

After splitting the dendrite into dendritic branches, we quantified the length of each branch to assess the spine density across layers (Supplementary Fig. S14a), we picked out the dendritic branches longer than 10 μm to ensure the accuracy of quantification. Our analysis revealed that spine density in layer 2/3 is significantly larger than the other two layers, which is consistent with previous research (Tjia *et al.* 2017). We further examined the density of different types of spine in each dendrite across layers Supplementary Fig. S15a, which shows that the density of thin/long thin types in layer 2/3 is significantly larger than layer 4, whereas the density of mushroom spines is similar between the two layers.

In addition, we found the dendritic radius in layer 5 to be significantly larger than the other two layers (Supplementary Fig. S14b), likely driven by the inclusion of thick apical dendrites. Therefore, for the quantification of spines within individual dendrites, we further divided dendrites into two groups based on their radius—thin or thick—yielding several interesting findings. The exact split point in our study is radius=367.3 nm. The average spine head volume in layer 5 is significantly larger than that in layer 4 for both thinner and thicker dendrites (Supplementary Fig. S16a), no significant difference was found between layer 2/3 and layer 4, consistent with our finding in Fig. 5b. In addition, we studied the head volume within each layer comparing thick and thin dendrites, but no clear difference can be found. Average spine neck length in layer 4 is consistently longer than the other two layers, regardless of dendrite thickness (Fig. 6j, N: L2/3 small: 13662, large: 2974, L4 small 14272, large: 2790, L5 small: 6617, large: 3727, Mean±SE, from left to right: 874.2 ± 5.264, 1012.0 ± 6.092, 706.8 ± 6.519, 857.4 ± 11.186, 975.9 ± 13.446, 752.6 ± 9.061, *t*-test), a trend that was also seen with head radius (Supplementary Fig. S16c). Notably, the ratio of head radius to neck radius in layer 4 was significantly larger than the other two layers for spines in both thinner and thicker dendrites (Supplementary Fig. S16d).

In terms of the tripartite structure analysis, our previous conclusion that layer 4 exhibits a larger astrocyte perimeter ratio remains valid for thinner dendrites (Fig. 6k, N: L2/3 small: 12663, large: 2828, L4 small 12996, large: 2524, L5 small: 5780, large: 3212, Mean±SE, from left to right: 0.326 ±0.0023, 0.348 ± 0.0025, 0.303 ± 0.0036, 0.333 ± 0.0048, 0.349 ± 0.0059, 0.307 ± 0.0051, *t*-test), as is the case for the pre-synaptic wrapping ratio (Supplementary Fig. S17a). Lastly, analysis of the ratio of astrocyte contact being at the spine head or neck (Supplementary Fig. S17c) indicates that layer 2/3 has a clearly larger contact ratio than the other two layers, possibly due to the higher density and a greater arborization, occupying a larger volume (Lanjakornsiripan *et al.* 2018). Finally, analysis of this ratio within each layer shows that frequency of astrocyte contact at the spine neck is always significantly smaller than the contact at the head.

## 4 Conclusion and discussion

The rapid advancements in EM imaging of large-scale brain regions have significantly expanded research opportunities, particularly in exploring the brain's intricate internal structure. A key area of interest is the functional interaction between neurons and glia and the relationship between structure and the function. Central to this investigation is the detailed compartment segmentation of neurons, notably the subdivision of dendrites into finer components such as dendritic spines, spine heads, and necks.

Traditional methods in neuroimaging have either focused predominantly on surface segmentation or applied heuristic approaches to volumetric segmentation. These methods often struggle with surface irregularities along dendrites or lack sensitivity to specific structural shapes. In our study, we address these challenges by integrating both surface and volumetric features in the segmentation process. With VSOT, we approached dendritic spine segmentation (level-1) by formulating them as min-cut problems, aiming for the smoothest possible segmentation surfaces. For level-2 segmentation, we optimized the combination of surface and volume features through a two-step process involving the identification of the shortest separating cycle followed by the search for the minimal surface cut. Both tasks are solved efficiently with network flow theory.

Moreover, the lack of quantitative evaluation often limited previous studies due to missing ground truth data. We counter this by developing a human-annotated ground truth dataset, which validated the significantly improved accuracy of our method. We quantified structural variations across different cortical layers in a public EM dataset. Our methodology is valuable for accessing public EM datasets with neuronal reconstructions, potentially advancing the biological research community's understanding of neuroanatomy.

Analysis of general dendritic and tripartite characteristics across cortical layers in the MICrONS dataset revealed several prominent trends. First, average spine neck length was significantly higher in layer 4 than either layer 2/3 or layer 5. Given that neck length is postulated to be a crucial mechanism for electrically isolating individual spines from the parent dendrite (Araya *et al.* 2014), the increased neck length in layer 4 may represent a method of filtering direct synaptic input from the thalamus to fine-tune sensory processing. This possibility is strengthened by the increased astrocyte contact ratio with synaptic clefts in this layer compared to the others, providing an additional level of regulation by reducing the potential for synaptic crosstalk (Bernardinelli *et al.* 2014). The spine head volume parameter was highest in layer 5, though there was a bimodal distribution, potentially reflecting morphological differences in the various projection neuron subtypes that populate this area. Taken together, these results support a previous report that spine neck length and head volume do not show a significant correlation and are likely differentially regulated, with distinct roles to play in paradigms of synaptic plasticity (Ofer *et al.* 2021). For distinct spine types, VSOT showed an overall trend of decreased density of long thin spines along the 2/3-4-5 axis, which was countered by an increasing gradient of mushroom spines. Such a distinction was not strongly evident before the advent of large-scale EM datasets and analytical tools such as VSOT, though we should note that this largely subjective method of dividing spine types into distinct categories may not fully capture the morphological (and, logically, functional) spectrum of these structures (Ofer *et al.* 2021).

This quantitative performance evaluation demonstrates that VSOT outperforms peer methods. The volume-surface optimization in VSOT significantly enhances the robustness of segmentation for both dendritic shafts and spines, as well as dendritic spine heads and necks. However, when evaluating performance across different spine types, we found that segmentation of stubby spines was less accurate than other types—although still superior to peer methods. This limitation arises from the absence of a distinct neck, which complicates modeling compared to mushroom or thin dendritic spines. While our model does not fully capture all stubby spine scenarios, future work can leverage the quantification results to refine their classification for improved segmentation. Another limitation of our method is its reliance on accurate neuron segmentation from EM data, which can sometimes be erroneous. Future work will explore using raw EM data to detect compartments, allowing tolerance or correction of minor segmentation errors in the public EM dataset for more accurate neuron-glia quantification. Additionally, memory and processing time are concerns for large-scale data segmentation. In practice (see Supplementary Section S3.6 in Supplementary Material), we reduced computation time by using fewer surface points without significantly impacting accuracy, thanks to volume-surface optimization. Chunk-wise computation can save time and memory and facilitate parallel processing, though optimal chunk size varies by dataset. In tests on the MICrONS dataset, we split the dataset into chunks with each containing around 800×800×300 voxels ($12.8\times 12.8\times 12\;\mu m^{3}$ with voxel size 16 nm×16 nm×40 nm), requiring occasionally merging chunks to prevent the cropping of dendritic spines at the border, which increased processing time. Enhancing efficiency for larger datasets is a future research direction.

## Supplementary Material

btaf220_Supplementary_Data
