# Supplementary material for: VSOT: volume-surface optimization for accurate ultrastructure analysis of dendritic spines
Source: Bioinformatics. 2025 Apr 24;41(5):btaf220. doi: 10.1093/bioinformatics/btaf220 (PMC12150773; doi:10.1093/bioinformatics/btaf220)
Supplement: btaf220_Supplementary_Data [file btaf220_supplementary_data.zip › VSOT_Supplementary_Material.pdf]

Supplementary materials to “VSOT: Volume-Surface Optimization for Accurate Ultrastructure Analysis of Dendritic Spines”

1 Overall framework 3

2 Supplemental Methods 4

2.1 Level-1 segmentation 4

2.2 Efficiency in obtaining the shortest separating cycle in level-2 segmentation 7

2.3 min-cut surface in level-2 segmentation 8

2.4 Surface score for level-2 segmentation 9

3 Experiment setup, parameter settings 11

3.1 Dataset and peer methods for testing the level-1 segmentation 11

3.2 Multi-layer dataset constructed from MICrONS dataset 15

3.3 Details on the dataset created for testing the level-2 segmentation 16

3.4 Parameter setting for testing the performance of the level-2 segmentation 16

3.5 Examples of dendritic spine heads with different sphericity 18

3.6 Time and accuracy using different parameters for our method on the test dataset 19

4 Score design for quantifying dendritic spines 20

5 More quantification results of dendritic spines 22

6 Score design for quantifying tripartite structures 23

7 More quantification results of tripartite structure 26

8 Segmentation of dendritic branches 28

9 Classification of the types of dendritic spine and quantification of dendritic branches 29

10 More quantifications related to dendritic structures 31

Reference 36

1 Overall framework

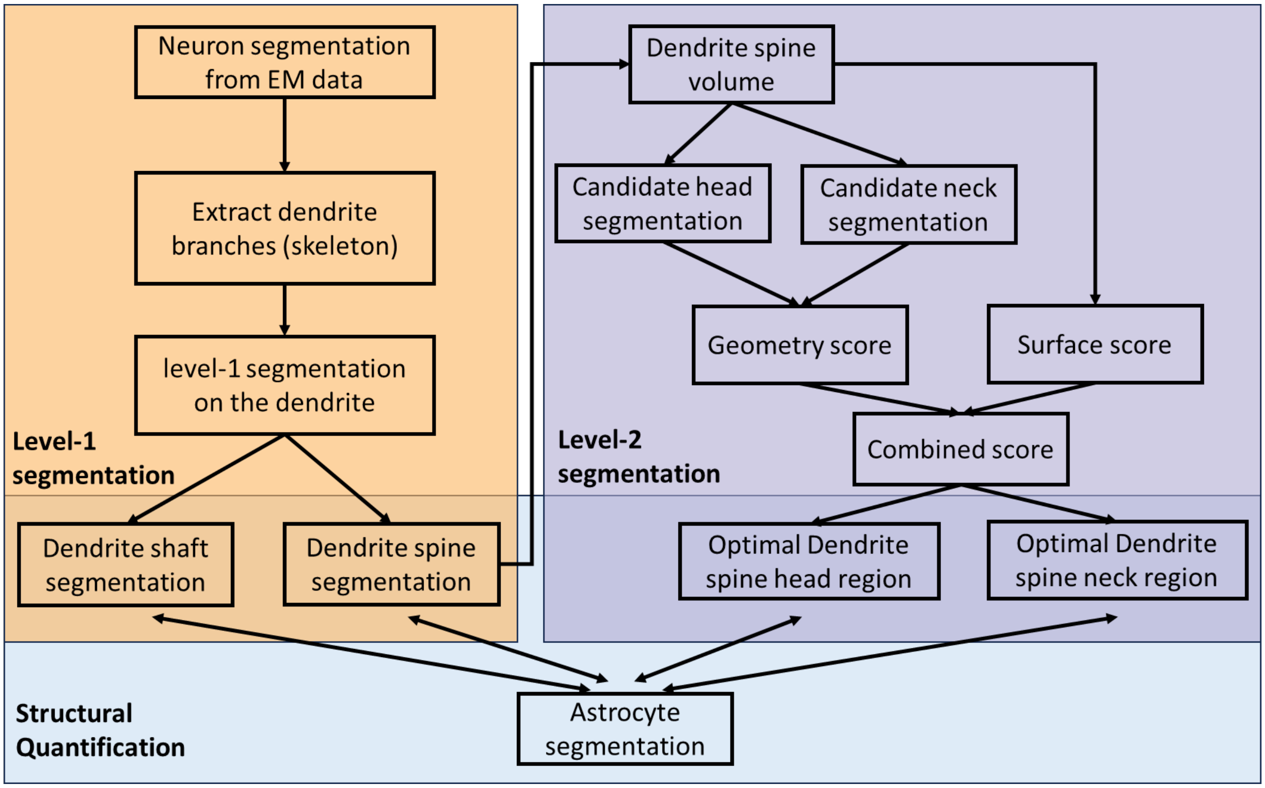

Fig. S1 Overview of the schema of VSOT. VSOT provides two levels of segmentation of the neuron reconstruction, which can be obtained from the EM data. For the level-1 segmentation, the dendritic spine and dendrite shaft are separated with a min-surface cut objective. Level-2 segmentation further segment the dendritic spine into dendritic spine head and neck. Based on the compartment segmentation of the dendrite, as well as the astrocyte segmentation, which can also be obtained from EM data, we can quantify the structure of the dendrite as well as the tripartite structure.

2 Supplemental Methods

2.1 Level-1 segmentation

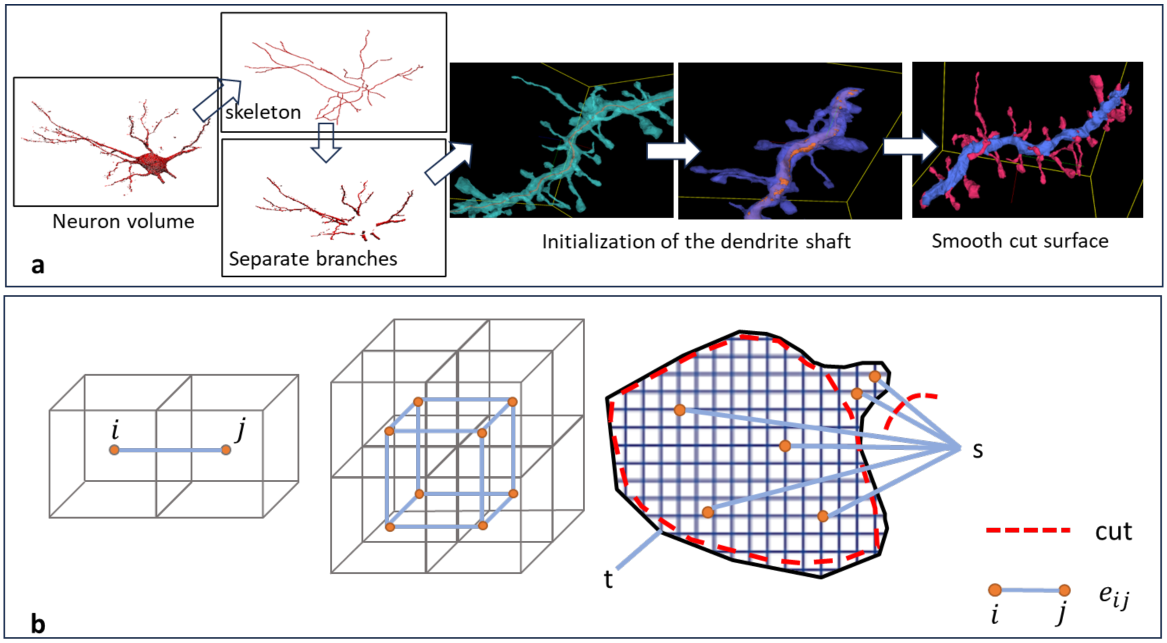

Fig. S2 (a) Workflow of our proposed level-1 segmentation method, which includes preprocessing, initialization and segmentation by finding the optimal cut surface between dendritic spine and shaft. (b) Graph design for solving the min-cut problem in level-1 segmentation.

As shown in Fig. S2 (a), the input to the whole pipeline is the volume of the neuron.

We first applied preprocessing so that the soma and axons were removed and so that we could obtain the independent neuronal branches with dendritic spines. In obtaining the soma region, we first obtained the skeleton using Kimimaro (Silversmith *et al.* 2021) and then calculated the distance to the boundary for each voxel along the skeleton. We picked out the center of the soma by searching for the local maxima, which gives the largest possible radius r for the soma. Next, the soma region was detected and removed by spherical fitting, such that any region within the range of $k \times r + b$ were removed, with k and b being two parameters to tune how large the

range we hope to remove. In our experiments, we set $k = 3$ and $b = 500\ (nm)$. After which, we worked on each branch.

The key to accurate segmentation relies on our newly designed model for the optimal dividing plane that distinguishes the dendritic spine from dendrite shaft. We modeled the dendrite shaft by minimizing its surface and, at the same time, minimizing the volume of its counterpart, which can be written as,

$$\min_{\mathcal{V}_s} Surface(\mathcal{V}_s) - \lambda \times \text{Volume}\ (\mathcal{V}_s) \tag{1}$$

where $\mathcal{V}_s$ is the region of the dendritic shaft, $Surface(\mathcal{V}_s)$ is the surface voxel number surrounding the volume region $\mathcal{V}_s$, and $Volume(\mathcal{V}_s)$ is the volume of the region $\mathcal{V}_s$. If we denote the region of dendritic shaft as a set of voxels S, we can re-write the objective mathematically as below,

$$\min_{S} |S - S^{o}| + \lambda |X - S|, S \in X \tag{2}$$

where X represents the overall voxel set, which is the volume of the whole dendritic branch. And S is the desired voxels of the dendrite shaft, S^{o} is the interior of the set S, and $S - S^{o}$ is the surface of the shaft. $X - S$ represents the voxels belonging to the dendritic spine. The objective is to minimize the size of the boundary of set S and the complement of S at the same time. Where λ is a parameter to control the balance between the two costs: the larger the λ, the more likely S will be close to X so that the maximum volume can be achieved; at the same time, the smaller the λ, the more likely S will be small in surface (also smaller in volume); an extreme case under this condition is that $S = 0$, which we obviously want to avoid. Directly optimizing this objective function is challenging, but we found it can be formulated into a min-cut/ max-flow problem which can be efficiently solved.

Based on the cost function Eq. (2), we constructed a graph $G_d = V_d, E_d$, where V_d are all the nodes of the graph, with each node corresponding to a voxel inside the dendrite. E_d is the undirected edge set, which contains all the edges linked between nearby voxels. In our graph design, we used six connectivity. We voxels as graph nodes, and also included two pseudo nodes s and t to represent the final two segmentation groups separately, which are the dendrite shaft and dendritic spine. Our graph is built in 3D, but for easier visualization, we can display our graph in 2D as Fig. S2 (b). Each node is connected to both its nearby nodes and to the pseudo-source node. In addition, the nodes belonging to the boundary of the volume are connected to the sink node. Based on the graph design we can obtain a solution of the minimum cut separating the nodes into source group (classified as dendrite shaft) or sink group (classified as dendritic spine).

To formulate the min-cut problem, define L as the labeling of the graph G, such that each node (other than the two pseudo nodes s and t) is assigned with a binary label $L(i)$. In our case, the binary label can be considered as the assignment of whether a voxel belongs to the dendrite shaft ($L(i) = 0$) or dendritic spine ($L(i) = 1$). The labelling can form an edge set $C = \{(i,j)|L(i) \neq L(j), (i,j) \in E_{dendrite}\}$. Cutting the corresponding edge set will give the sum cut $cut(C) = \sum_{(i,j)\in C} w(i,j)$. Where $w(i,j)$ is the weight on the edge between node i and j. Based on the graph construction, we can express the min-cut problem as

$$\min_{L} g(L) = \min_{L_1 \dots L_N} \sum_{n\in Nei(m)} cut(L_m, L_n) + \lambda \sum_{n=1}^{N} cut(L_n) \quad (3)$$

where $cut(L_m, L_n)$ accounts for the cut between two nearby nodes. When both nodes belong to the same group, the cut will be 0; otherwise, the cut will be the edge weight $w(i,j)$. Any cut between the nodes will lead to adding one unit in the surface in the final dendrite shaft. So, the

first part represents the total surface area of the final dendrite shaft. While $cut(L_n)$ defines the cut of the link between any node and the source node. For example, if voxel n belongs to the dendrite shaft, then $cut(L_n) = 0$, otherwise $cut(L_n) = 1$. With source node s included, $cut(L_n)$ can also be interpreted as $cut(L_n, L_s)$, and L_s will always be 0. Any cut will lead to a unit increase in the volume of the dendritic spine so that the second part of the Eq. 3 equals total volume of the dendritic spine. During the implementation, we set the weight $w(i,j) = \sqrt{dist(i)dist(j)}$, where $dist(i)$ is the geodesic distance to the skeleton of the shaft. And λ is set as $\sqrt[3]{(resx * resy * resz)^2}/32$ which considers the resolution of x, y and z dimensions. Thus, by solving the min-cut problem, we can obtain the optimized segmentation of the dendritic spine, balancing both the volume and the surface.

2.2 Efficiency in obtaining the shortest separating cycle in level-2 segmentation

The reason why we can reduce the theoretical complexity of solving the shortest separating cycle is largely due to the design of the new dual graph, which converts the min-cut formulation into finding the shortest path in a planar graph. Since it has been theoretically proven that the complexity of solving the shortest path in the planar graph can be $O(N)$ based on r-separators, where N is the total number of vertices (Henzinger *et al.* 1997), which is much faster than solving a min-cut problem. Therefore, the complexity for each point can be reduced from $O(N^3)$ to $O(N)$ thanks to this conversion, and the total complexity is reduced from $O(N^4)$ to $O(N^2)$.

In addition, we found that by opening the 3D surface-based graph through the shortest path between source and sink, the graph constructed from the planar graph can directly work with all the vertices on the surface; in other words, the graph can remain fixed when searching for the shortest separating cycle for different points. This is because any separating cycle must go

through the shortest path ST between two ends S and T. (1) For the points along this path, the shortest separating cycle can be directly obtained by searching the shortest path between the point to its twin point on the twin path $S'T'$. (2) On the other hand, for a point A not on that shortest path, suppose the shortest separating cycle intersects with the path ST at the point B. Thus, by finding the pair of points B on ST and B' on the twin path $S'T'$ that are closest to A, we can obtain the two path segments BA and AB'. Combining these two paths will form the shortest separating cycle in the original 3D graph.

2.3 min-cut surface in level-2 segmentation

Specifically, in our graph design, each node corresponds to one tetrahedron, and since there is a triangle shared by any pair of adjacent tetrahedrons, two adjacent nodes are linked by an edge. The edge weight is defined as the area of the shared triangle. As illustrated in the Fig. 2 (e), two tetrahedrons $BCOE$ and $BCOG$ are defined as two graph nodes i and j separately, the edge e_{ij} is linked between the two with the edge weight set as $weight(e_{ij}) = Area(BCO)$. In this way, minimizing the cut is equivalent to minimizing the surface area, which is the area-sum of all the intersected triangles. To solve the s-t min-cut, we must define a pseudo-source node s representing the head part and one pseudo-sink node t representing the sink part. Since we already obtained the candidate dividing cycle, we can cut the surface into the head and neck parts, as shown in the right bottom of Fig. 2(e). By forcing the nodes with the head surface part connected to the source i.e., connected to the pseudo source with weight $weight(si) = \infty$, and the nodes with the neck surface part connected to the sink, i.e., connected to the pseudo sink with weight $weight(ti) = \infty$. We can force the cut to go through the dividing cycle. After finding the minimum cut, all the tetrahedrons connecting to the source will be considered the head part, and

the rest will be considered the neck part. Additionally, by grouping the tetrahedra into two parts, we can delineate the cut surface by identifying the overlapping triangles between the two tetrahedron sets. Based on this, the surface of the head part can also be obtained. In our implementation, for an efficient computation, we use the bounding box of the separating cycle to limit the computational units used for finding the min-surface cut.

2.4 Surface score for level-2 segmentation

Since the curvature is sensitive to local change, we applied Taubin smoothing (Taubin 1995), which can smooth the surface without shrinkage. Following surface smoothing, we calculate the principal curvature using the method from (Rusinkiewicz 2004), which entails first determining face-wise principal curvatures and then computing vertex-wise principal curvatures by averaging those of adjacent faces. The face curvature is estimated from the second fundamental tensor defined based on the directional derivatives of the surface normal,

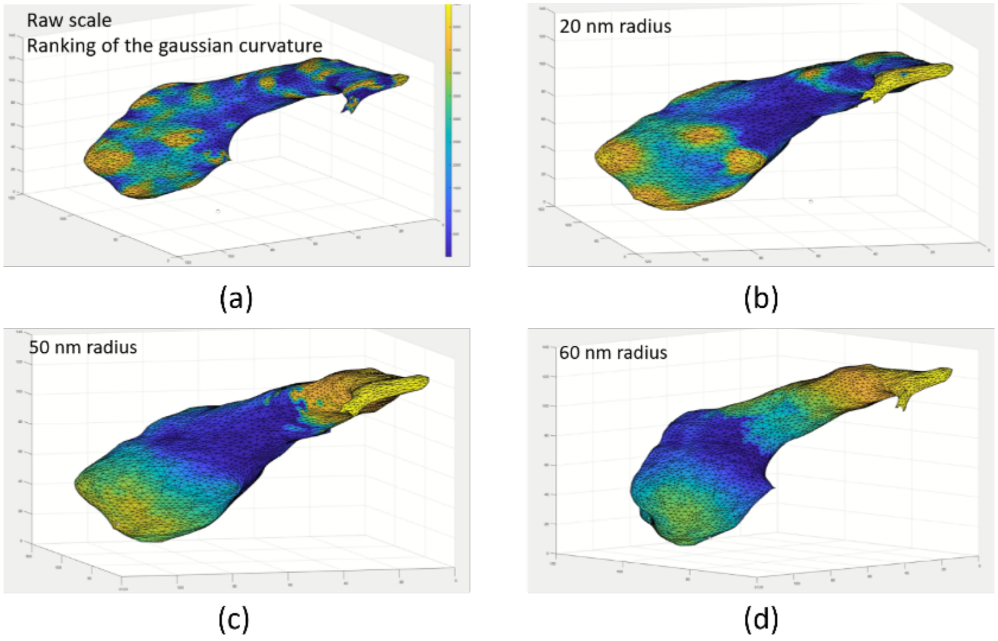

Fig. S3 Surface score map obtained at each point with different settings on the window size.

$$RN_2 = (D_u n D_v n) = \begin{pmatrix} \frac{\partial n}{\partial u} u & \frac{\partial n}{\partial v} u \\ \frac{\partial n}{\partial u} v & \frac{\partial n}{\partial v} v \end{pmatrix} \tag{4}$$

where u and v are the orthogonal directions. Multiplying the tensor with any vector in the tangent plane gives the derivative of normal at that direction. So, for each triangle, three equations can be obtained for each vector between a pair of terminals, and RN_2 can be estimated based on the minimum least square. Then, the two principal curvatures are obtained from the eigen decomposition of RN_2.

Since this method (Rusinkiewicz 2004) calculates the principal curvature based on the nearest neighbors of the target vertex, it cannot deal with the curves at different scales. We designed a way to calculate the curvature with tunable window size to adapt to different scales. The basic idea is to search for all the vertices within the radius of the targeting vertex and re-mesh the local surface so that the size of each triangle will be enlarged. As shown in Fig. S3, we can see that the difference around the head-neck dividing line is more prominent as we enlarge the window size for the target dendritic spine.

3 Experiment setup, parameter settings

3.1 Dataset and peer methods for testing the level-1 segmentation

To quantify the accuracy of the dendritic spine segmentation, we proceed with the performance testing based on the manual segmentation of (Kasthuri *et al.* 2015). We selected the manual annotation of 344 dendritic spines for the largest dendritic region (dendrite 5), which contains five branches and one apical dendrite (https://lichtman.rc.fas.harvard.edu/vast/Kasthuri2015_Segmentation_vss.zip).

In total, we obtained the volume segmentation of six branches, as shown in Fig. S4. Utilizing this data, we generated the mesh surfaces using the iso2mesh package (Tran, Yan and Fang 2020). Next, we create a ground truth dataset for segmenting the dendritic spine. This involved assigning the labels of the spine/shaft to the faces on the surface by checking each face's nearest voxel in the volume segmentation.

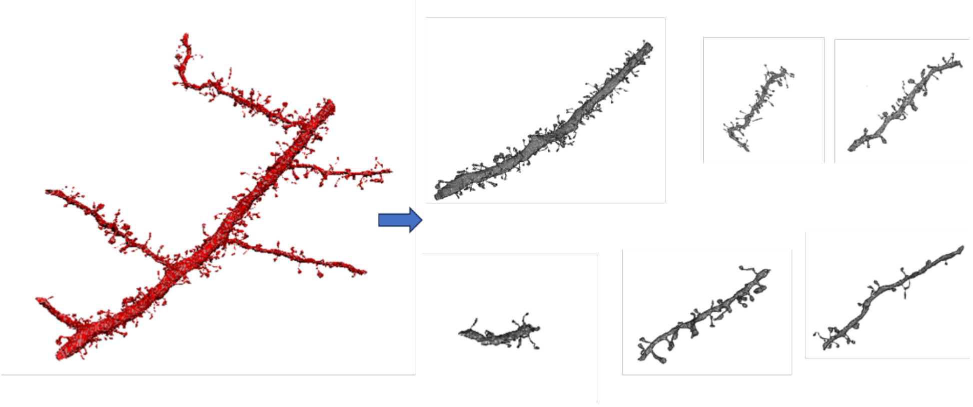

Fig. S4 The dataset we curated from the public dataset, which contains five branches and one apical dendrite.

NEURD (Celii *et al.* 2023) and SpineTool (Ekaterina *et al.* 2023) employ surface segmentation techniques. The former is based on the initial over-segmentation of the surface and then follows several rules for filtering. These rules were designed based on the signed distance function (SDF)

and other surface and volume features. A challenge encountered with this software was the complexity involved in tuning parameters. The process can be very time-consuming since it has more than ten parameters required to tune. NEURD (https://github.com/reimerlab/NEURD) works by first generate spine candidates and then filter using a series of rules. We found two dominating parameters 'clusters_threshold' and 'smoothness_threshold' in the first part. Then, using the best combination of these two parameters, we tune the other two dominating parameters in the second part, which are 'spine_n_face_threshold_bare_min' and 'spine_sk_length_threshold_bare_min'. The parameter list giving optimal F1 score is shown in Table S1, and we then populated the parameters to the remaining samples. SpineTool (https://github.com/spbstu-applied-math/SpineTool.Software) is based on the thresholding of the distance to the skeleton for each surface point. Similar to NEURD, we also fine-tuned the parameter of SpineTool based on the first sample of the dataset, the two parameters are set as **(1) sensitivity: 0.001, (2) correction: -3**. The third method (Michalska *et al.* 2023) (named "Morph" in the main body and following) is based on volume segmentation. It isolates dendrite shaft regions by removing dendritic spine necks through morphological operation. In the experiment, we found eroding the structure by a spherical kernel with **radius = 4** with a spherical kernel **radius = 2** as dilation can give the best results.

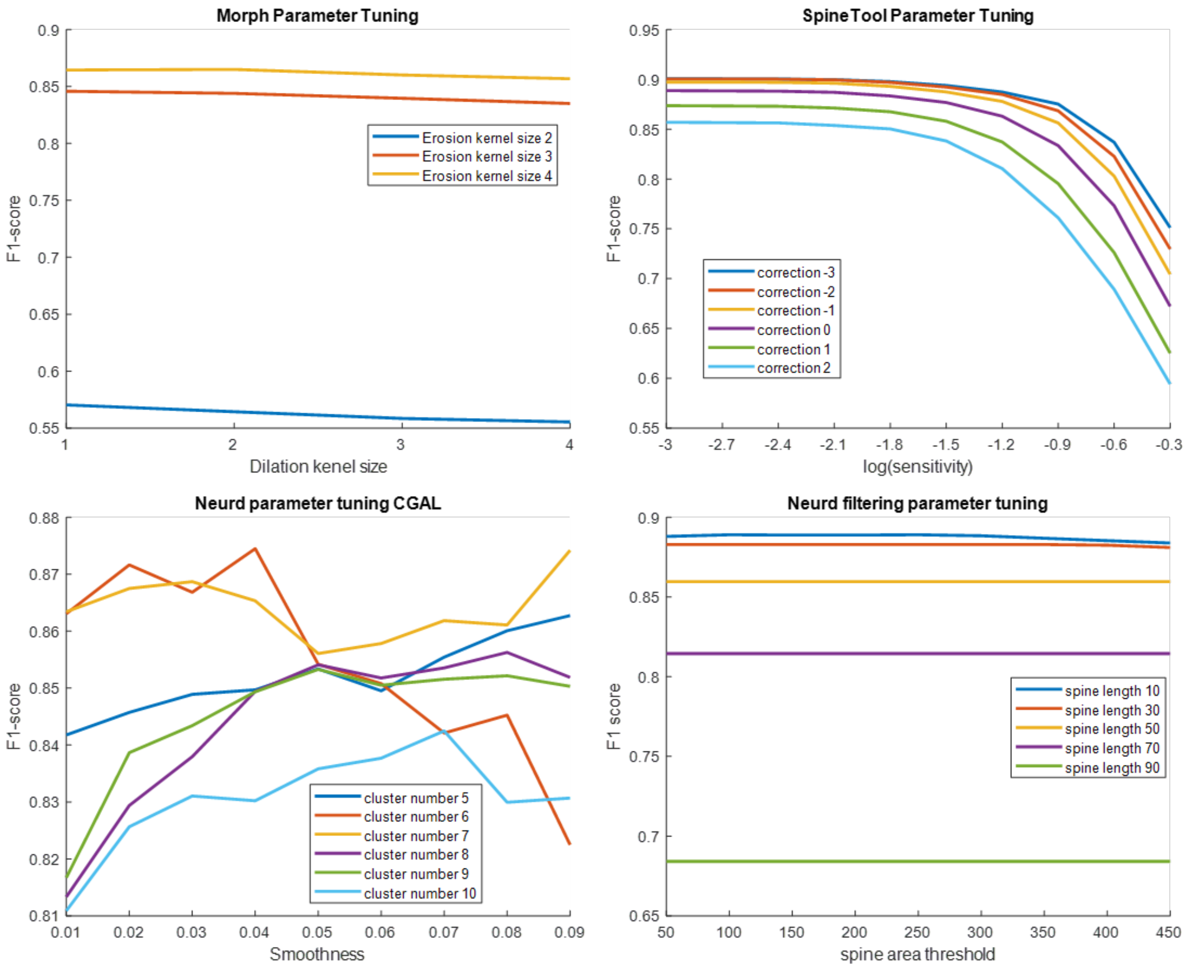

Fig. S5 Parameter tuning for the peer methods

Table S1 Parameter settings used in NEURD for the dendrite segmentation

soma_vertex_nullification	False
skeleton_endpoint_nullification	False
clusters_threshold	6
smoothness_threshold	0.04
shaft_mesh_volume_max	10000
shaft_close_hole_area_top_2_mean_max	100000
shaft_mesh_n_faces_min	10
spine_n_face_threshold_bare_min	100
spine_sk_length_threshold_bare_min	10
filter_by_volume_threshold_bare_min	0.01
bbox_oriented_side_max_min_bare_min	0.4
sdf_mean_min_bare_min	0.002
spine_volume_to_spine_area_min_bare_min	0.00002s

3.2 Multi-layer dataset constructed from MICrONS dataset

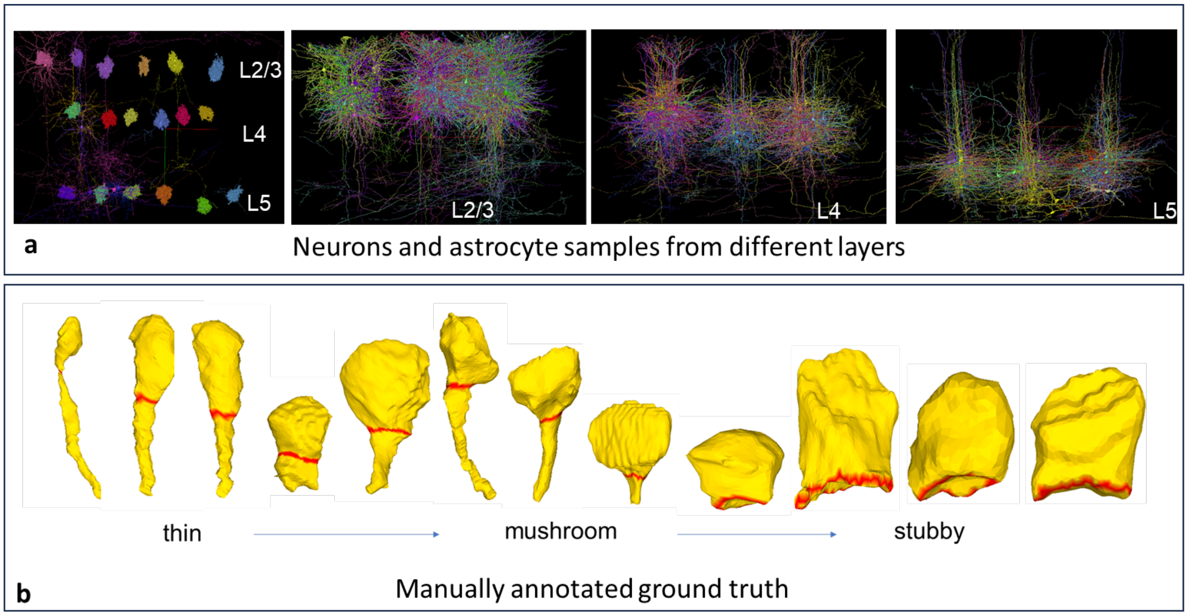

Fig. S6 (a) Selection of the neurons from different visual cortex layers from the MICrONS dataset. (b) Our manually annotated dendritic spine segmentation covers various types of dendritic spine.

The selection process is centric towards the astrocyte, since we intended to quantify the structural relationship between astrocyte and neuron. The decision of which layer is based on the examples given by the MICrONS dataset as well as the manual examination of the neuronal structure.

3.3 Details on the dataset created for testing the level-2 segmentation

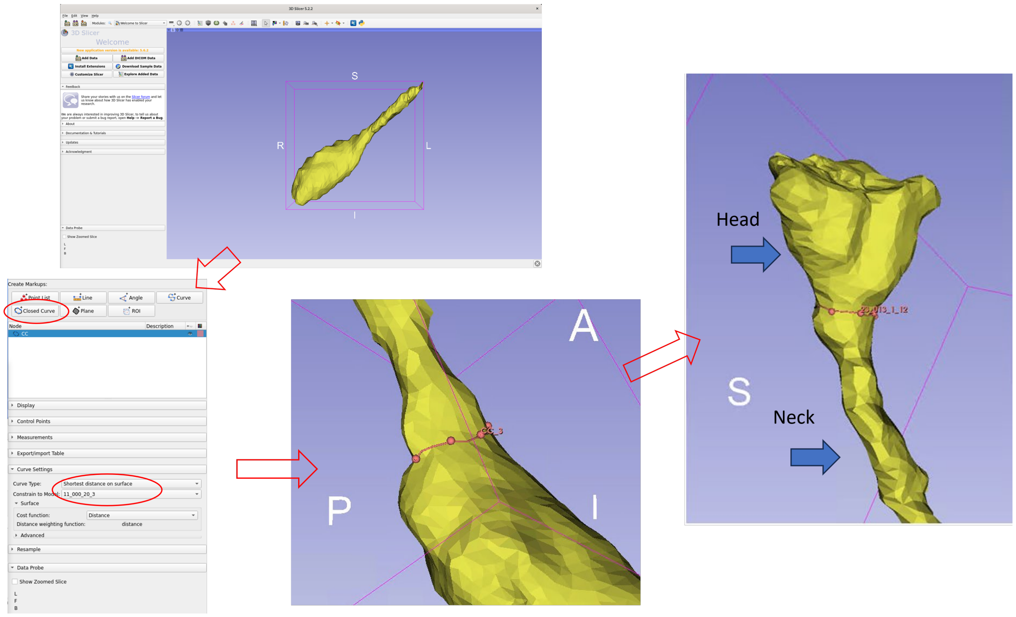

Fig.S7 Manual annotation to constructing the dataset for testing level-2 segmentation. The closed curves are manually drawn in the 3D slicer software using the Markups functions. Notably, in the curve settings, we set the points on the curve linked through the shortest distance on the surface.

For each mesh surface file, we manually annotated the best line segments to be the cut between dendritic spine head and neck. The part belonging to "head" part should be following a spherical structure. The other part, "neck" should be close to a pipe-like structure. We drew the curves by setting the curve to be linked by shortest path along the surface as shown in Fig.S7.

3.4 Parameter setting for testing the performance of the level-2 segmentation

Three peer methods were selected for comparing the performance of level-2 segmentation.

1. O.N method (Ofer *et al.* 2021): we downloaded the scripts from https://github.com/NTCColumbia/Ultrastructural-analysis-of-dendritic-spine-head-and-neck-. It required the library CGAL for calculating the Shape Diameter Funciton (SDF) value as well as the distance to the skeleton ($Dist$) for each point on the surface. We used CGAL-5.5.2 for this test. We set parameters 'number_of_clusters' = 2,

‘smoothing_lambda’ = 0.1, the combined scoremap of SDF and $Dist$ was defined as $score = SDF + (Dist - min(Dist))/(max(Dist) - min(Dist))/2$. Since the output of this method is the label assignment for each triangle formed by three edges, in order to obtain the cut edge between the head and neck part, we designed a method to search for the boundary in the post processing. In certain cases, there can be more than one cycles of edges, where the shortest one was selected for the final output. Then we can obtain the segmentation of head and neck.

2. D.S method (Dorkenwald *et al.* 2022) : decides the cutting cycle on the surface based on the distance to the skeleton. Following the rules designed in the corresponding paper. We first generated the skeleton using kimimaro (https://github.com/seung-lab/kimimaro; Silversmith and Wu, 2022) and extracted the distance to the segment boundary (DB) for each point on the skeleton. Then we determined the direction from spine-head to shaft by comparing the average DB of the points on each half of the skeleton. Next, we identified anchor 1 (the point with minimum distance to the segment boundary (DB)) and anchor 2 (the point with the maximum DB) and decided the best skeleton node for splitting based on the criteria described in the paper. Which starts the scan from the closest node to anchor 2 that had DB value less than $\frac{1}{3}DB_{anchor1} + \frac{2}{3}DB_{anchor2}$. Finally, the triangles that are close to the skeleton nodes on the head side are considered to be head surfaces, while the rest considered as neck surfaces.
3. T.H method (Tamada *et al.* 2020): this paper decides the separation of the surface by finding the point where the significant change of the cross-section locates. The original paper utilized the NeuroMorph library in Blender to produce the cross-section of

dendritic spines, which we later found hard to run in batch processing. Thus, we reimplemented the idea based on the Trimesh library (https://trimesh.org/). The output of the best circle contains only the intersection points of the cross-section and the surface. In order to match the circle to the complete cycle on the surface, we further wrote a script to find the closest vertices to these intersection points and then link them into a complete separating cycle (cut). After obtaining the separating cycle, we then separated the surface into head and neck part.

3.5 Examples of dendritic spine heads with different sphericity

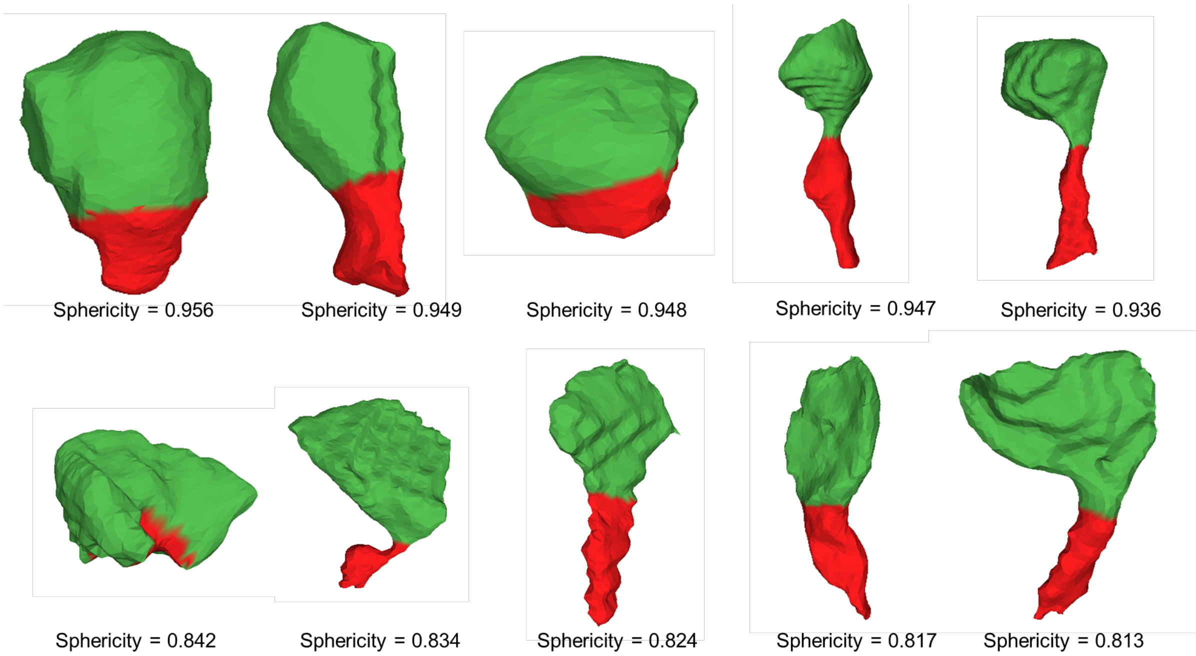

Fig. S8 Examples of the dendritic spine head segmentation result: head (green), neck (red). The larger the sphericity score (closer to 1), the closer the head to a spherical structure.

3.6 Time and accuracy using different parameters for our method on the test dataset

Table S2 time spent per sample vs accuracy under different initial candidates

#initial candidates	Time (seconds)	IOU
**1200**	**32.91**	**0.8923**
400	18.47	0.8909
100	12.79	0.8912
80	10.68	0.8911
40	10.66	0.8819
20	10.60	0.8740

In implementing the level-2 segmentation, we can avoid calculating the surface score and geometry score for all the points on the surface. One way to save time would be changing the parameter which is the top-N points with smallest surface score as initial candidates, which then be used for the computation of geometry score. We tested VSOT using different parameter settings on 100 mushroom dendritic spines with eight cores running computation in parallel. As shown in Table S4, we can see a relatively small number for a still satisfactory result. Even though the accuracy decreases with fewer initial candidates, as shown in Table S2, the accuracy does not drop much. For example, using 100 initial candidates would cost 32.3% of the time spent to achieve the best result, but the accuracy IOU was decreased by only 0.0012%.

4 Score design for quantifying dendritic spines

We quantified five scores. Most of these metrics are straightforward and easily derived from our volumetric segmentation. During the quantification, we removed the outliers (top and bottom 1%) which can be caused by wrong segmentation or extreme cases.

(1) Volume of the dendritic spine head: Using compartment segmentation, we achieve binary segmentation of both the dendritic spine head and neck. Each point in the segmentation represents one volumetric unit. Based on the resolution of the segmentation which is 16 nm for both the XY direction and 40 nm for the Z direction, each unit represents 10240 nm^3.

(2) Max cross-section area of the dendritic spine head: A challenge in quantifying the dendritic spine head's cross-section area is deciding the axial direction. Considering that the molecules are absorbed through the synaptic cleft, the gradient of the flow should be vertical to the cleft. Thus, it would be meaningful to examine the maximum area that is vertical to the flow. So, we define the cross sections as the point sets that are parallel to the synaptic cleft. By applying PCA on the 3D coordinates of the cleft region, we identify the two principal directions. By transforming the coordinate such that the two main directions of the cleft region are mapped to the X and Y directions, aligning the Z-axis with the axial direction, we can traverse through Z direction and evaluate the area of each XY plane. In this way, we can obtain the maximum area of the dendritic spine head.

(3) Length of the Dendritic Spine Neck: Defining the neck's length requires first determining its centerline. We first obtain the two furthest points of the structure and then find the path between them such that they go through the voxels most distant from the boundary. Given the identified junctions between the dendrite shaft and spine and the junctions between the dendritic spine head

and neck, centroids of the junctions were used as these two furthest points. Subsequently, within the inverted 3D transform map, we identified the shortest path connecting these points. In this way, we can find the 3D centerline, and the length can also be calculated by summing all the distances between adjacent pairs of points.

(4) Mean radius of the cross-section of the dendritic spine neck. We group all the voxels along the centerline of the neck and check the widths of the spine neck at each voxel, which is measured as their nearest distance to the boundary.

(5) SD of the radius of the cross-section of the dendritic spine neck: With the widths obtained from (4), we can also quantify the standard deviation of the widths. This score measures how close the dendritic spine neck is to a cylindrical structure.

5 More quantification results of dendritic spines

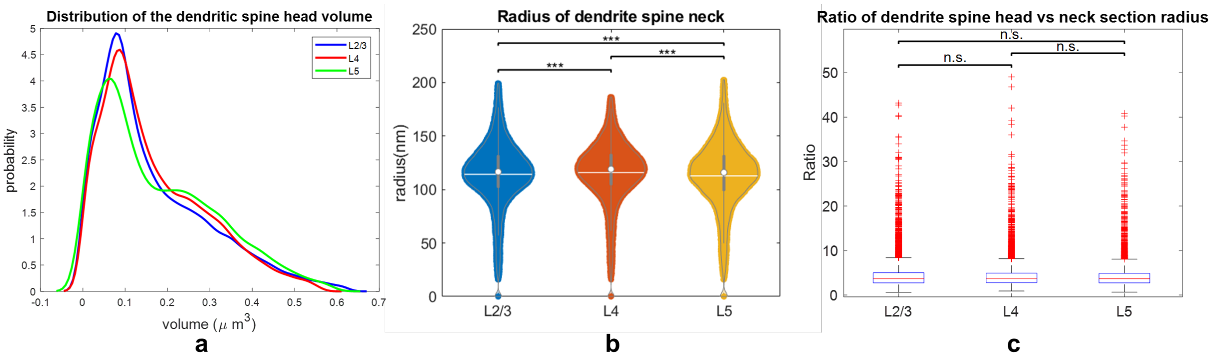

Fig. S9 More quantification results of dendritic spines. (a) The distribution of dendritic spine head volume of L5 shows a clear bimodal shape compared to L2/3 and L4. (b) The neck radius of layer 4 is on average larger than layer 2/3 and layer 5(N:L2/3, L4, L5: 13766,13436,7958 Mean ± SE: 1.14e+02±0.27, 1.16e+02±0.25, 1.13e+02±0.37 t-test), which can be due to the bi-directional communication required in layer 4. (c) No significance difference was found between layers for the ratio of head/neck radius (N: 13766, 13436, 7958,Mean ± SE: 2.84±0.0088, 2.96±0.0093, 2.77± 0.0112,t-test).

6 Score design for quantifying tripartite structures

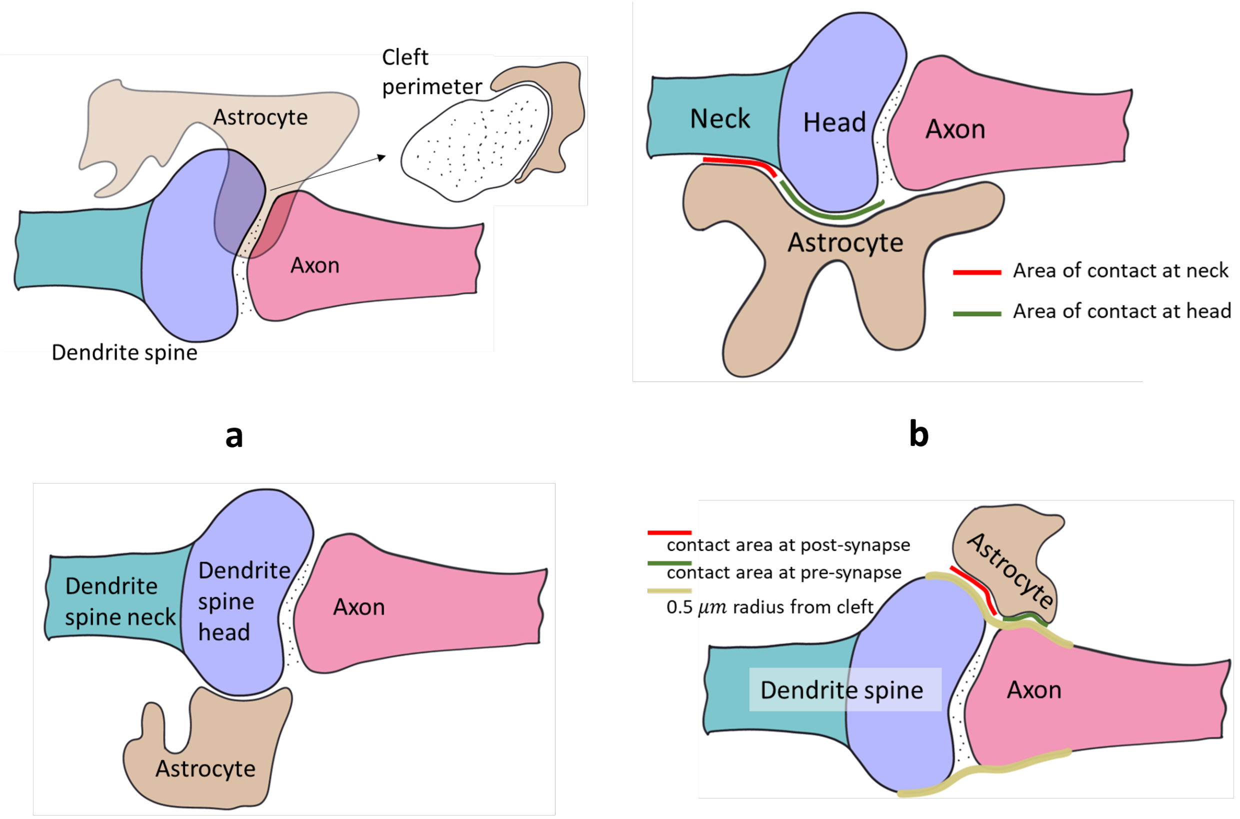

Fig.S10 Illustration of the score designs for quantifying the tripartite structure. (a) Perimeter wrapping ratio. (b) Ratio between the astrocytic contact area at head and neck part of the dendritic spine. (c) The ratio of the dendritic spines that are in contact with astrocyte at head or neck. (d) Contact ratio at pre/post synaptic region, defined as the ratio of astrocytic contact area within the 0.5 μm radius from the perimeter of the synaptic cleft.

One key function of the astrocyte is to uptake glutamate in the extracellular space, which is through receptors like EAATs. It is hypothesized that these receptors are predominantly located just outside the synaptic cleft to maximize the astrocyte's sensitivity to changes in glutamate spillover, given this region's high dynamic response range. To quantify the tripartite structure, our focus was on assessing the extent of astrocyte envelopment around the synapse.

(1) the perimeter wrapping ratio: As depicted by Fig.S10 (a), we measured how much of the cleft is directly in contact with the astrocyte. To quantify this, we focus on the perimeter of the cleft and check the ratio of it being in contact with the astrocyte. In the dataset, the presynaptic axonal region and postsynaptic dendritic spine are in direct contact. Therefore, we defined the cleft region as the intersection of the presynaptic and postsynaptic regions, expanded by one layer in all directions. The cleft boundary was determined by excluding the presynaptic and postsynaptic regions from this intersection. A full coverage of the synaptic cleft yields a score of 1, while no coverage results in a score of 0.

(2) Ratio of the astrocyte contact area between the head and neck: We focused on the dendritic spine and quantified the number and the ratio of the astrocytic contact on either the spine head or the spine neck. The ratio is defined as the ratio of the number of voxels at astrocyte-head contact and the number of voxels at astrocyte-neck contact. As shown in Fig.S10 (b).

(3) Ratio of the dendritic spines that are contact with astrocyte at head or neck: Group analysis of the dendritic spines, as to the ratio of dendritic spines that are in contact with any astrocyte region. Furthermore, since we are able to segment the dendrite into spine head and neck, we can further check where the contact is located. As shown in Fig.S10 (c).

(4) Pre/ Post synaptic wrapping ratio: As shown in Fig.S10 (d). In addition to the astrocyte region that is directly in contact with the synaptic cleft, we also quantify the astrocytic coverage in the vicinity of the cleft. And since the average distance between nearby PSD is around $0.5\mu m$ (Medvedev *et al.* 2014). so that we search within the radius of $0.5\mu m$ from the perimeter of the cleft. Given the dense packing of cells with neurons, the glutamate's free space is considerably smaller than a sphere of radius $0.5\mu m$. We defined the free space as the boundary along both the presynaptic and postsynaptic surfaces. From the perimeter of the cleft region, we search along

the surface of pre-synapse and post-synapse that is within the range of $0.5\mu m$ in terms of geodesic distance. Within these free spaces on either pre-synapse or post-synapse, we quantify the number and the ratio of voxels that are in contact with the astrocyte.

(5) Weighted astrocytic contact area: The previously described quantifications assume uniform influence of all astrocyte surface points on glutamate. However, this might not be the case since the concentration of the escaped glutamate decreases the further it goes away from the cleft. Accordingly, (Medvedev *et al.* 2014) employed an exponential decay model for glutamate concentration, positing that uptake efficiency is proportional to this concentration. Thus, each astrocyte surface point at distance r is assigned a weight defined by

$$w(r) = \exp(-18r^2) \tag{5}$$

where r is measured in micrometers. In our analysis, we grouped all the astrocyte surface points that are in the free space ($0.5\mu m$ range) and used the sum of the weight to obtain the weighted area as a new score.

$$S_w = \sum_{i=1\cdots N} w(r_i) \tag{6}$$

During the quantification, we removed the outliers (top and bottom 1%) which can be caused by wrong segmentation or extreme cases.

7 More quantification results of tripartite structure

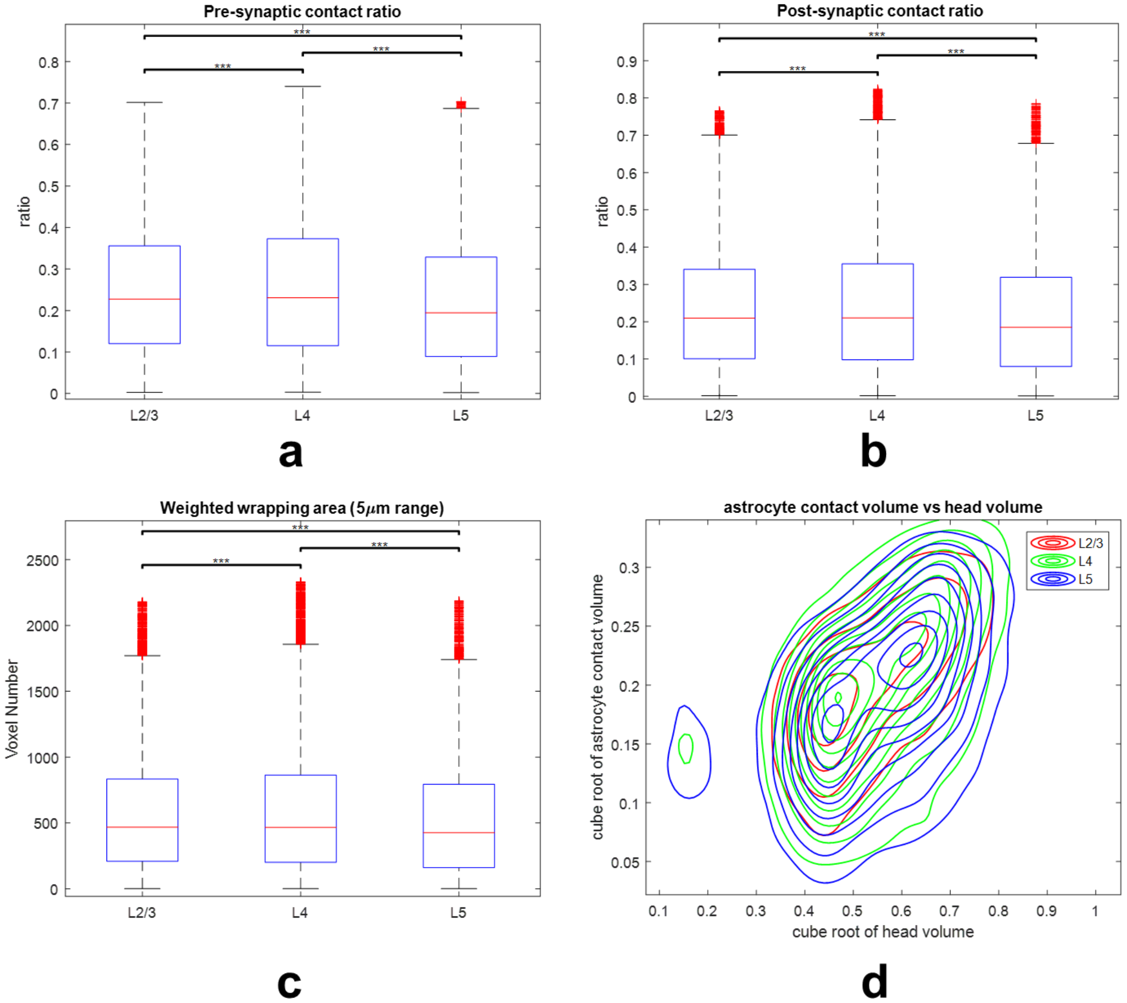

Fig. S11 More quantification results of the tripartite structure. (a-b) Dendritic spines in layer 4 have larger astrocytic contact ratio at both pre-synapse and post-synapse. (a N:L2/3, L4, L5: 13898, 12899, 6855, mean±SE: 2.48e-01±1.4e-03, 2.56e-01±1.5e-03, 2.24e-01±2.0e-03, b: N: 14040, 13082, 7160, mean±SE:2.36e-01±1.4e-03, 2.45e-01±1.6e-03, 2.19e-01±2.0e-03 ,t-test) (c) Similarly, after incorporating the distant astrocyte voxels that are not directly in contact with dendritic spine, the weighted wrapping area around the cleft is also larger in layer 4. (N:14530, 13613,7457,mean±SE:5.73e+02±3.85, 5.95e+02±4.33, 5.36e+02±5.36 t-test)(d) There is a linear relationship between the cube root of astrocytic contact volume and the cube root of head volume of the synaptic cleft volume. Interestingly, in layer 4 & 5, the bimodal pattern can be discovered from the distribution of two features.

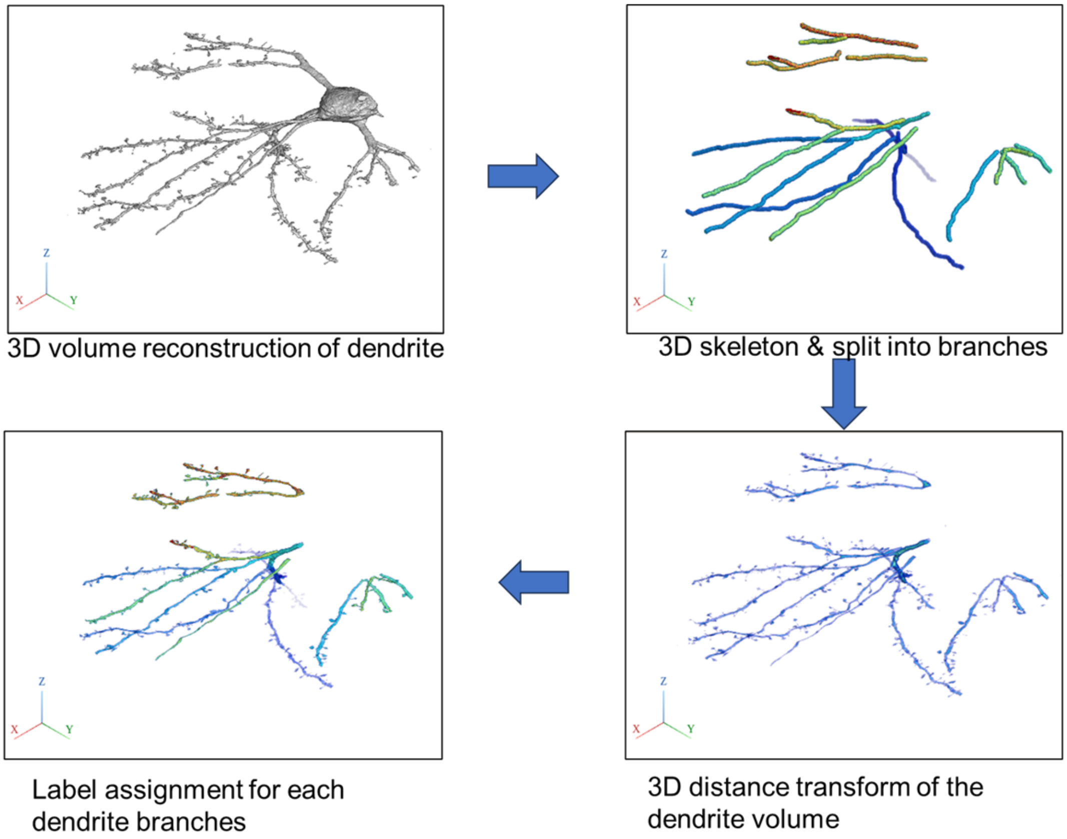

Fig. S12 The workflow for the segmentation of dendritic branches. Using the skeleton branches as seeds, we grow them based on the distance transform score map of the dendrite volume. In this way, we can separate the dendrite shaft into separate branches and further assign the label to the dendritic spines on each branch.

8 Segmentation of dendritic branches

To quantify the structure of individual dendritic branches, including dendritic spines and tripartite structures, we segmented the dendritic structure into separate branches.

As shown in Fig. S12, starting with the skeleton of each dendrite, we identified and removed branch points to divide the skeleton into distinct branches. Each branch then served as a seed for growth within a 3D distance map using the watershed algorithm. This approach allowed us to separate and label the dendritic shafts of different branches individually. Subsequently, we labeled the dendritic spines for each branch based on their affinity to the corresponding dendritic shaft.

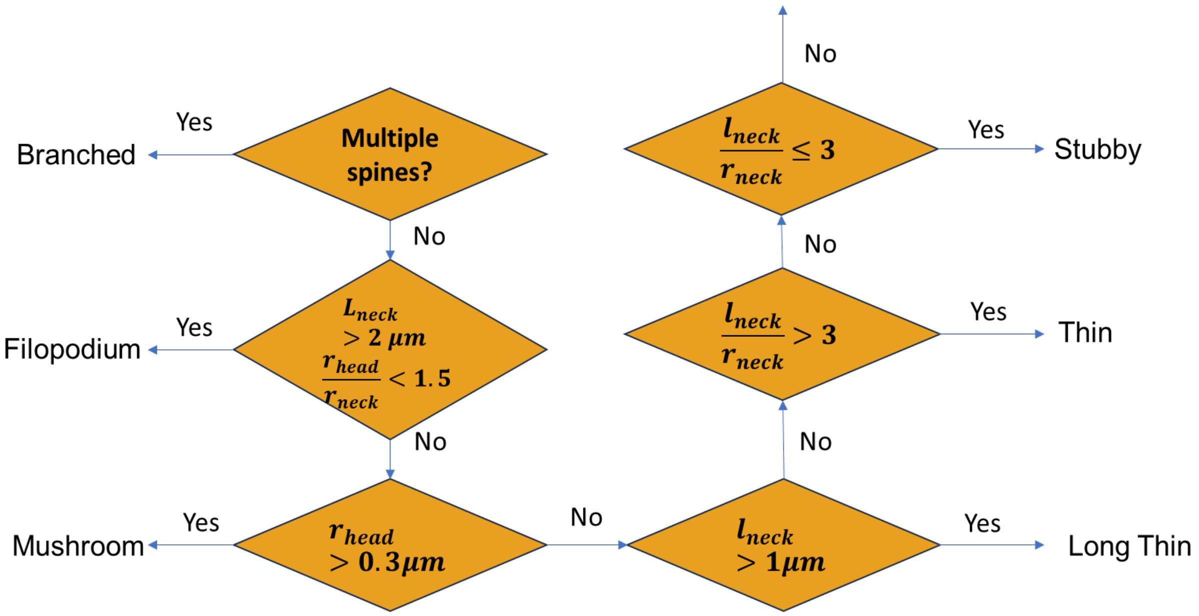

Fig.S13 Decision tree for the classification of dendritic spines.

9 Classification of the types of dendritic spine and quantification of dendritic branches

The classification of dendritic spines was based on criteria similar to those established by (Risher *et al.* 2014); the decision tree is shown in Fig. S13.

(1) Branched: Defined as more than one spine head attached to a single spine neck. However, due to segmentation inaccuracies in the MICrONS dataset, some dendritic spines were erroneously connected to each other, to axons, or even looped back to connect to the dendritic shaft at two points. To filter out these artifacts and accurately identify true branched spines, we removed any spine with two connections to the dendritic shaft. We then defined potential branched spines as those with more than one long branch (≥20 voxels in a 16x16x40 nm resolution) in the skeleton. Given the limited number of branched spines, we further designed a GUI so that we can manually review all candidates to eliminate incorrect classifications due to segmentation errors.

(2) Filopodium: neck length > 2 μm and the ratio of the head radius/ mean neck radius < 1.5

(3) Mushroom: the mean head radius > 0.3 μm

(4) Long thin: neck length > 1μm

(5) Thin: neck length/ mean neck radius > 3

(6) Stubby: neck length/ mean neck radius <= 3

The quantification of dendritic branches was categorized as follows:

(1) Dendritic branches alone: the length, mean radius of each dendritic branch was quantified.

(2) Spines on the dendrite:

a. Dendritic spine density (per μm)

b. Dendritic spine type score. In addition to quantifying the density of each type of dendritic spine, we further defined a weighted density of each type representing the average maturity of dendritic spines on each dendritic branch. In which, we picked the types Filopodium, Long thin, Thin and Mushrooms. The weights assigned to each type are ¼, ½, ¾, 1. Which represents the maturity of each type. The reason why we discarded the types stubby and branched is because we found there are controversies related to the stubby and branched spines in terms of aging (Helm *et al.* 2021; Barzó *et al.* 2024). But to ensure the fairness of comparison between dendrites with different degrees of discarded spines, we replaced the discarded spines as the mean type based on the mean score of the included ones.

c. Mean dendritic spine head volume/radius

d. Mean dendritic spine neck radius/length

e. Mean ratio between the dendritic spine head radius and neck radius

f. Mean astrocyte wrapping ratio at cleft perimeter/pre-synapse/post-synapse

g. Mean binary contact ratio/ratio of the contact area between the astrocytic contact at the head and neck.

10 More quantifications related to dendritic structures

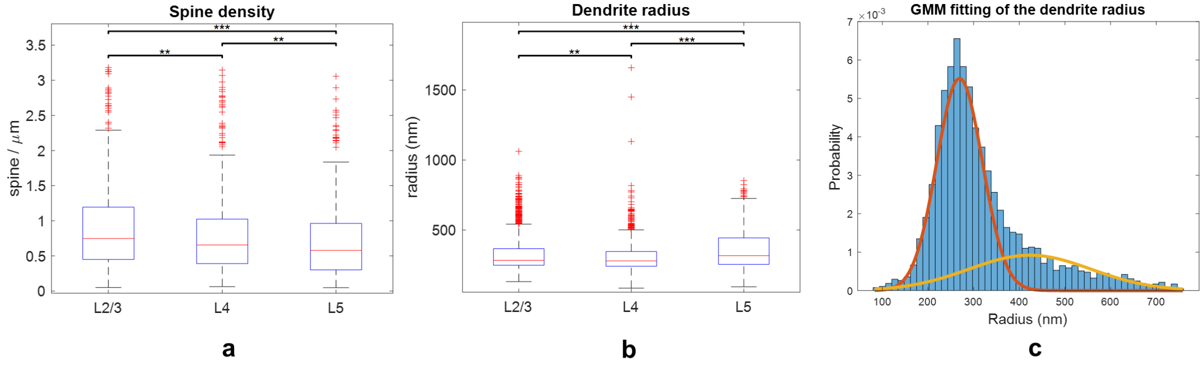

Fig. S14 (a) The spine density in layer 2/3 is significantly larger than the other two layers (N:521, 613,422, mean±SE: 8.94e-01±2.81e-02, 7.90e-01±2.33e-02, 7.07e-01±2.70e-02, Mann-Whitney U test)(b) The dendrite radius, on the other hand, does show significant differences for layer 5, which is largely due to the inclusion of the thick apical dendrites.(N:1927, 2275, 1434,mean±SE: 2.73e+02±3.39, 2.48e+02±2.95, 2.88e+02±4.64, Mann-Whitney U test) This prompted the idea of splitting the dendrites into thin group and thick group. (c) The split was done by fitting a GMM model on all the mean radius of dendritic branches. The division point was then decided based on the Bayesian decision with priors set as equal.

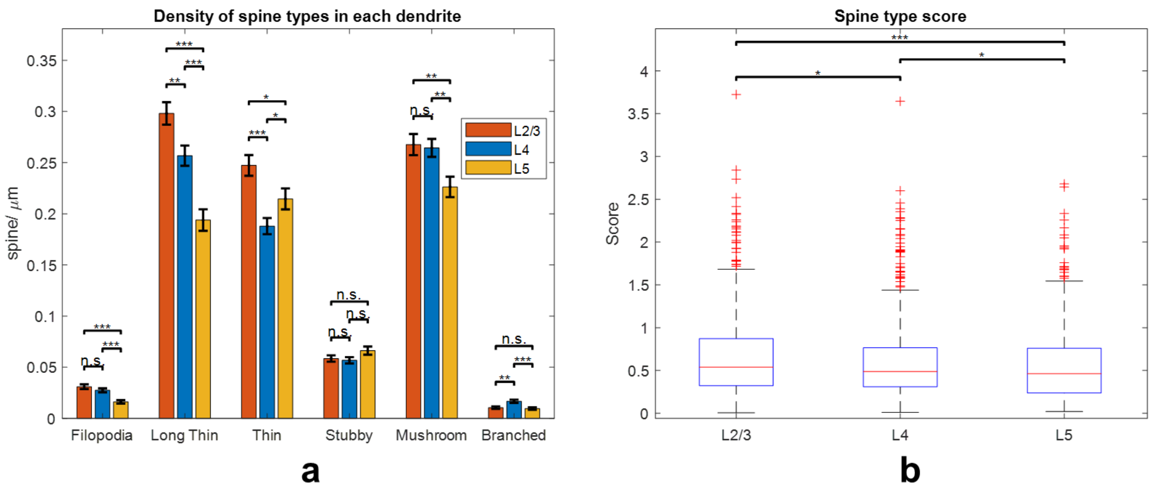

Fig. S15 (a) Spine density in different groups in each dendritic branch across three layers. Compared between layer 2/3 and layer 4, the thin & long thin types are more prominent in layer 2/3, but there was not much difference in the density for the mushroom type. In addition, shallower layers also show a significantly larger density in filopodia type of spine.(N: 526, 618, 420, Mean±SE, from left to right L2/3, L4, L5, Filopodia:0.031±0.0023,0.028±0.0020, 0.016±0.0017 , LongThin: 0.298±0.0110, 0.257±0.0099, 0.194±0.0105, Thin: 0.247±0.0101, 0.188±0.0079, 0.215±0.0103, Stubby: 0.059±0.0031, 0.057±0.0031, 0.066±0.0040, Mushroom: 0.268±0.0103, 0.264±0.0088, 0.226±0.0099, Branched:0.011±0.0012, 0.017±0.0015,0.010±0.0012 , Mann-Whitney U test) (b) Spine type score across three layers. Possibly due to the higher spine density in shallower layers, the spine type score in layer 2/3 is larger than layer 5. (N:526, 618, 420, Mean±SE: 6.66e-01±2.23e-02, 6.01e-01±1.84e-02, 5.57e-01±2.19e-02, Mann-Whitney U test)

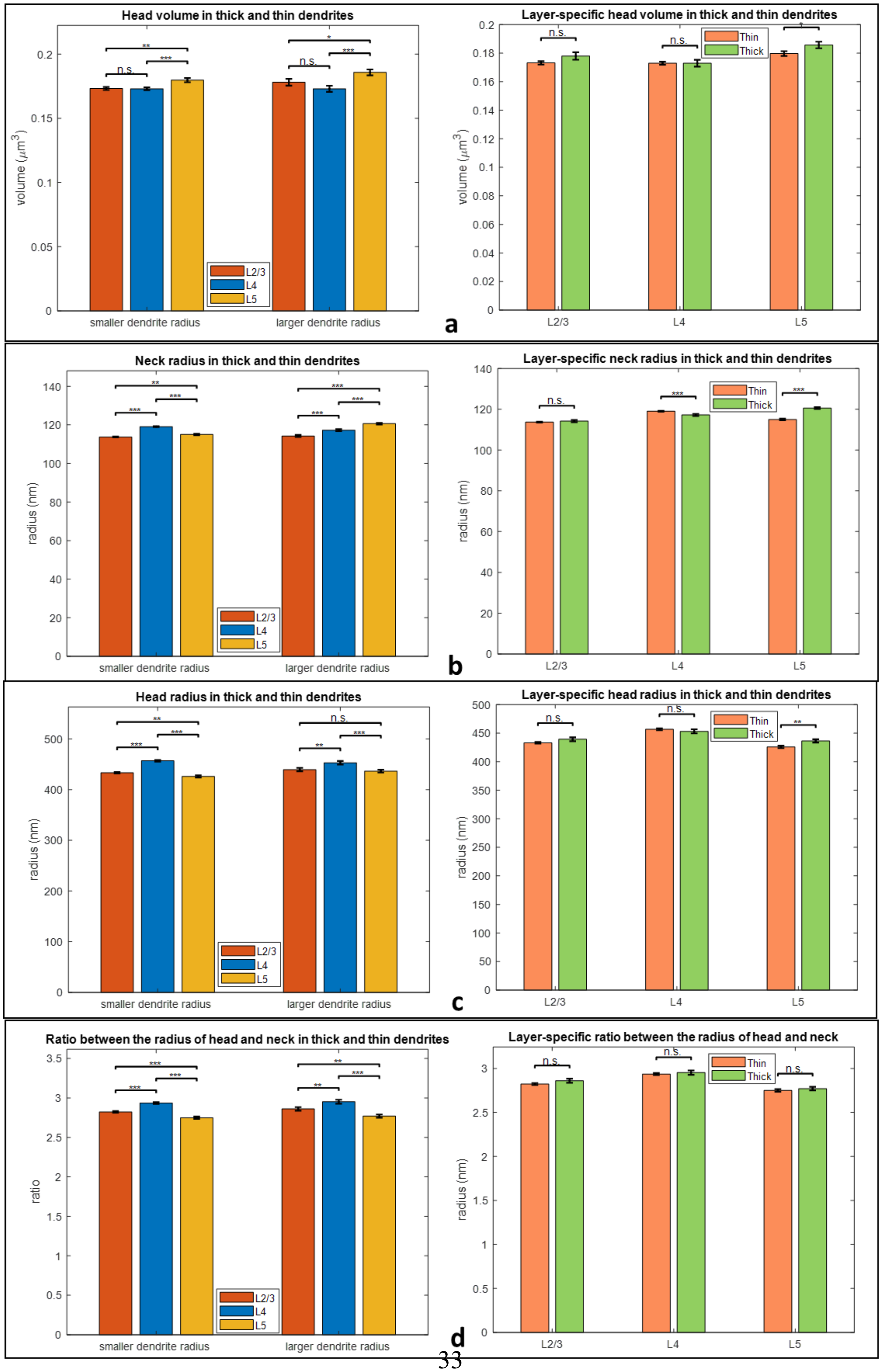
Head volume in thick and thin dendrites
Layer-specific head volume in thick and thin dendrites
Neck radius in thick and thin dendrites
Layer-specific neck radius in thick and thin dendrites
Head radius in thick and thin dendrites
Layer-specific head radius in thick and thin dendrites
Ratio between the radius of head and neck in thick and thin dendrites
Layer-specific ratio between the radius of head and neck
volume (μm^3)
radius (nm)
ratio
smaller dendrite radius
larger dendrite radius
L2/3
L4
L5
Thin
Thick
n.s.
a
b
c
d

Fig. S16 (a) The average volume of the dendritic spine head is significantly larger in layer 5 than the other two layers. When separating the dendrites based on the radius, the head volume of spines in layer 5 still have significantly larger head volume than the other two layers in both thinner and thicker dendrites. No significant difference was found between thinner and thicker dendrites in each layer. (N:L2/3 small: 14798, L2/3 large 3214, L4 small 15363, L4 large 3005, L5 small 7489, L5 large 4116, Mean±SE, from left to right, L2/3 small, L4 small, L5 small, L2/3 large, L4 large, L5 large: 0.173±0.0012,0.173±0.0011,0.180±0.0017,0.178±0.0026,0.173±0.0024,0.186±0.0023, t-test) (b-c) For both the head radius and neck radius, layer 4 has a larger value than layer 2. When examining the thick dendrites, layer 4 has significantly larger head radius than layer 5. Interestingly, in layer 2/3 and layer 4, thick dendrites have significantly larger neck radius than thin dendrites. But in layer 5, the head radius of the thick dendrites is significantly larger.(b: N:L2/3 small: 13566, L2/3 large 4156, L4 small 14873, L4 large 3640, L5 small 7790, L5 large 3698, Mean±SE, from left to right: L2/3 small, L4 small, L5 small, L2/3 large, L4 large, L5 large: 113.72±0.234,119.07±0.217,115.02±0.372,114.21±0.524,117.28±0.500,120.61±0.4527, t-test, c: N:L2/3 small: 9201, L2/3 large 2821, L4 small 9394, L4 large 2345, L5 small 4984, L5 large 2331, Mean±SE, from left to right: L2/3 small, L4 small, L5 small, L2/3 large, L4 large, L5 large,433.27±1.55,456.86±1.62,426.06±2.09,439.45±3.38,452.97±3.55,436.53±2.91, t-test) (d) The larger ratio between head radius and neck radius is significantly larger in layer 4 for the thin dendrites. But the difference within each layer is not significant.(N:L2/3 small: 9428, L2/3 large 2067, L4 small 9229, L4 large 1836, L5 small 4372, L5 large 2403, Mean±SE, from left to right: L2/3 small, L4 small, L5 small, L2/3 large, L4 large, L5 large: 2.823±0.0106, 2.936±0.0111, 2.749±0.0151, 2.861±0.0229,2.952±0.0251,2.771±0.0203, t-test)

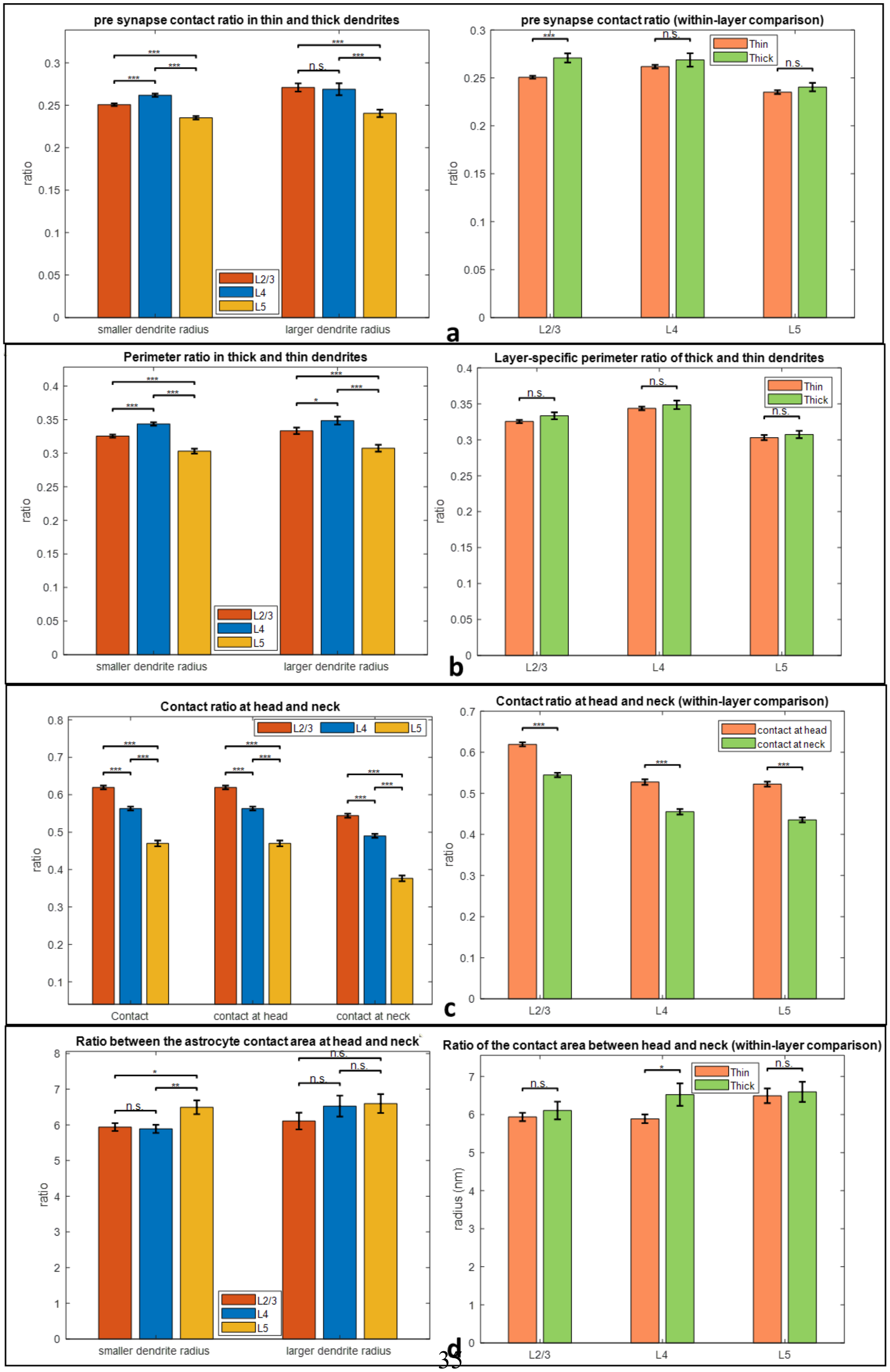

pre synapse contact ratio in thin and thick dendrites
pre synapse contact ratio (within-layer comparison)
ratio
smaller dendrite radius
larger dendrite radius
L2/3
L4
L5
Thin
Thick
n.s.
a
Perimeter ratio in thick and thin dendrites
Layer-specific perimeter ratio of thick and thin dendrites
b
Contact ratio at head and neck
Contact ratio at head and neck (within-layer comparison)
Contact
contact at head
contact at neck
c
Ratio between the astrocyte contact area at head and neck
Ratio of the contact area between head and neck (within-layer comparison)
radius (nm)
d

Fig. S17 (a-b) Comparison of the astrocytic wrapping ratio at pre-synapse and within the perimeter of the dendrite cleft. Layer 4 shows significantly larger score than the other two layers for the thin dendrites. Other than layer 2/3, the difference between the thick and thin dendrites in each layer is not that significant. (a: N:L2/3 small: 13723, L2/3 large 3046, L4 small 14041, L4 large 2735, L5 small 6256, L5 large 3467, Mean±SE, from left to right, L2/3 small, L4 small, L5 small, L2/3 large, L4 large, L5 large:0.251±0.0018,0.262±0.0019,0.226±0.0028,0.259±0.0037,0.262±0.0045,0.235±0.0039, t-test, b: same as Fig.6(k) N: L2/3 small: 12663, large: 2828, L4 small 12996, large: 2524, L5 small:5780, large: 3212, Mean±SE, from left to right, L2/3 small, L4 small, L5 small, L2/3 large, L4 large, L5 large: 0.326±0.0023, 0.348±0.0025, 0.303±0.0036, 0.333±0.0048, 0.349±0.0059, 0.307±0.0051, t-test)(c) The ratio of dendritic spines in each dendritic branch that have astrocyte in contact with head part or neck part of the dendritic spine. Layer 2/3 clearly shows a significantly larger ratio. Furthermore, the contact is prone to be at head part for all the layers.(N:L2/3 contact: 1258, L2/3 contact at head 1258, L2/3 contact at neck 1258, L4 contact 1333, L4 contact at head 1333, L4 contact at neck 1333, L5 contact 882, L5 contact at head 882, L5 contact at neck 882, Mean±SE, from left to right L2/3 contact, L4 contact, L5 contact, L2/3 contact at head, L4 contact at head, L5 contact at head, L2/3 contact at neck, L4 contact at neck, L5 contact at neck: 0.619±0.0050, 0.563±0.0051,0.470±0.0077,0.679±0.0050,0.563±0.0051,0.470±0.0077,0.544±0.0053,0.490±0.0052,0.377±0.0075, t-test) (d) For the thin dendrites, the ratio between the astrocyte contact area in head part and neck part is significantly larger in layer 5 than other two layers. But within each layer, the score is not much different between the thin and thick dendrites.(N:L2/3 small: 7021, L2/3 large 1678, L4 small 6526, L4 large 1343, L5 small 2446, L5 large 1395, Mean±SE, from left to right, L2/3 small, L4 small, L5 small, L2/3 large, L4 large, L5 large:5.938±0.1090,5.888±0.1135,6.493±0.1928,6.106±0.2333,6.525±0.2954,6.597±0.2638, t-test)

Reference

Barzó P, Szöts I, Tóth M *et al.* Electrophysiology and Morphology of Human Cortical Supragranular Pyramidal 1 Cells in a Wide Age Range 2. 2024:1–26.

Celii B, Papadopoulos S, Ding Z *et al.* NEURD: A mesh decomposition framework for automated proofreading and morphological analysis of neuronal EM reconstructions. *bioRxiv* 2023:2023.03.14.532674.

Dorkenwald S, Li PH, Januszewski M *et al.* Multi-Layered Maps of Neuropil with Segmentation-Guided Contrastive Learning. *bioRxiv* 2022:2022.03.29.486320.

Ekaterina P, Peter V, Smirnova D *et al.* SpineTool is an open-source software for analysis of morphology of dendritic spines. *Sci Rep* 2023;**13**:1–14.

Helm MS, Dankovich TM, Mandad S *et al. A Large-Scale Nanoscopy and Biochemistry Analysis of Postsynaptic Dendritic Spines*. Springer US, 2021.

Henzinger MR, Klein P, Rao S *et al.* Faster Shortest-Path Algorithms for Planar Graphs. *J Comput Syst Sci* 1997;**55**:3–23.

Kasthuri N, Hayworth KJ, Berger DR *et al.* Saturated Reconstruction of a Volume of Neocortex. *Cell* 2015;**162**:648–61.

Medvedev N, Popov V, Henneberger C *et al.* Glia selectively approach synapses on thin dendritic spines. *Philos Trans R Soc B Biol Sci* 2014;**369**:1–6.
Michalska JM, Lyudchik J, Velicky P *et al. Imaging Brain Tissue Architecture across Millimeter to Nanometer Scales*. Springer US, 2023.
Ofer N, Berger DR, Kasthuri N *et al.* Ultrastructural analysis of dendritic spine necks reveals a continuum of spine morphologies. *Dev Neurobiol* 2021;**81**:746–57.
Risher WC, Patel S, Kim IH wa. *et al.* Astrocytes refine cortical connectivity at dendritic spines. *Elife* 2014;**3**:1–24.
Rusinkiewicz S. Estimating curvatures and their derivatives on triangle meshes. *Proc - 2nd Int Symp 3D Data Process Vis Transm 3DPVT 2004* 2004:486–93.
Silversmith W, Bae JA, Li PH *et al.* Kimimaro: Skeletonize densely labeled 3D image segmentations. 2021, DOI: 10.5281/zenodo.5539913.
Tamada H, Blanc J, Korogod N *et al.* Ultrastructural comparison of dendritic spine morphology preserved with cryo and chemical fixation. *Elife* 2020;**9**:1–15.
Taubin G. Curve and surface smoothing without shrinkage. *IEEE Int Conf Comput Vis* 1995:852–7.
Tran AP, Yan S, Fang Q. Improving model-based functional near-infrared spectroscopy analysis using mesh-based anatomical and light-transport models. *Neurophotonics* 2020;**7**:1.
